# A 1 MDa protein complex containing critical components of the *Escherichia coli* divisome

**DOI:** 10.1038/srep18190

**Published:** 2015-12-08

**Authors:** Erik N. Trip, Dirk-Jan Scheffers

**Affiliations:** 1 Department of Molecular Microbiology, Groningen Biomolecular Sciences and Biotechnology Institute, University of Groningen, The Netherlands

## Abstract

Cell division in bacteria is an essential process that is carried out at mid-cell by a group of cell division proteins referred to as the divisome. In *Escherichia coli*, over two dozen cell division proteins have been identified of which ten are essential. These division proteins localize sequentially and interdependently to the division site, after which constriction eventually produces two daughter cells. Various genetic and biochemical techniques have identified many interactions amongst cell division proteins, however the existence of the divisome as a large multi-protein complex has never been shown. Here, we identify a 1 MDa protein complex by native page that contains seven essential cell division proteins (FtsZ, ZipA, FtsK, FtsQ, FtsB, FtsL, and FtsN). The 1 MDa complex is present in rapidly dividing cells, but absent when cultures enter the stationary growth phase. Slight overexpression of the *ftsQ D237N* mutation that blocks cell division prevents formation of this 1 MDa complex. In cells depleted of FtsN, the 1 MDa complex is not assembled. Combined, our findings indicate that a large protein complex containing many different cell division proteins indeed exists. We note that this complex is very fragile and sensitive to the expression of tagged versions of FtsQ.

Bacteria procreate by cell division. This carefully orchestrated process involves elongation of the cell, constriction of the cell envelope, septum formation and finally fission. Cell division is carried out at mid-cell position by a group of cell division proteins referred to as the divisome, a name first coined by Nanninga^1^,^2^,^3^,^4^. Cell division is essential for bacterial life, therefore the genes and proteins involved in this process are conserved among most bacterial families^5^. Some of the bacterial division proteins, like the tubulin homologue FtsZ, are also present in Archeae, mitochondria and plastids^6^,^7^,^8^,^9^.

Over two dozen cell division proteins have been identified in the Gram-negative model bacterium *Escherichia coli*; ten of these (FtsZ, -A, -K, -B, -L, -N, -Q, -I, -W and ZipA) are essential and are considered as the core players of division^10^ (Fig. 1). In *E. coli*, cell division proteins localize sequentially and interdependently to the division site in the following order: FtsZ> [ZapA, FtsA, ZipA]> [FtsE, FtsX]> FtsK> [FtsQ, FtsB, FtsL]> FtsW> FtsI> FtsN> AmiC> EnvC (proteins arriving simultaneously in brackets, integral membrane proteins -IMPs- underlined)^11^. The localization sequence consists of two main steps^12^: the first step is the formation of a so-called proto-ring at mid-cell position, composed of FtsZ, FtsA and ZipA^13^. The proto-ring may provide the contractile tension needed for constriction^4^,^14^,^15^,^16^,^17^ and functions as an active operating platform targeted by other cell division proteins^18^. Formation of the proto-ring at mid-cell position is negatively controlled the Min-system, SlmA, and OpgH, that together prevent FtsZ-ring formation at cell poles, over the duplicating chromosome, and when the cell’s nutritional status is not (yet) favorable for division^19^,^20^,^21^,^22^. Proto-ring assembly is promoted by ZapA, ZipA and FtsA, that stabilize bundles of polymerized FtsZ and tether these bundles to the inner membrane^23^,^24^,^25^,^26^. Assembly of the proto-ring is followed by a ‘delay’-period of approximately 20% of the duration of the cell cycle, after which the other division proteins localize to the proto-ring (although timing of FtsE/X and FtsK is not exactly determined)^12^. During the delay, the appearance of constriction cannot be observed, indicating that actual division has not yet started and the delay allows DNA segregation to be completed^27^. After segregation of the chromosomes, which is most likely signaled through FtsK^28^,^29^,^30^, other proteins localize to the division site, such as FtsQ,-B, -L, -W, -I, -N, AmiC and EnvC upon which constriction commences^12^. Most of these ‘second step’ proteins are involved in septal peptidoglycan-synthesis and –hydrolysis.

Impairment or even overexpression of one protein in the interdependent sequence described above can cause other downstream proteins to fail to localize to the division site. Interestingly, when a downstream division protein is moved upstream by fusing it to an upstream protein it is able to back-recruit other upstream proteins and division can proceed, resulting in viable cells^11^. These findings provide hints towards the existence of the divisome as a protein super-complex or a temporary assembly that is comprised of all cell division proteins. However, until now strong evidence for the existence of such a complex remained elusive.

Thus far, interaction networks between divisome proteins have been investigated extensively with the use of various genetic and biochemical techniques. An extensive Bacterial Two Hybrid (BACTH) study found that FtsZ interacts with FtsA, ZipA, FtsK; FtsW interacts with FtsL, FtsN, FtsI; FtsL interacts with FtsK, FtsQ, FtsW; FtsI interacts with FtsA, FtsQ, FtsN, FtsI; and that FtsQ interacts with FtsK, FtsI, FtsN, FtsL^31^. Another, independent BACTH study confirmed that most Fts proteins are able to interact with multiple partners, as found in the first study^32^. Furthermore, FtsA dimerization and FtsA-FtsZ interaction as well as interactions between FtsN and FtsA, FtsI and FtsQ and interactions between FtsQ and FtsA, and FtsI were found. The interactions found with FtsN and FtsQ as bait were unexpected as they were between early and late recruits to the septum and *in vivo*, the latter interactions require the intermediate proteins FtsB, FtsL, FtsI and FtsW^32^. Förster Resonance Energy Transfer (FRET) resolved direct divisomal protein interactions such as FtsZ-FtsZ and FtsZ-FtsA^33^. Strikingly, ZapA also interacted with FtsN and FtsI, whereas FtsN interacted with itself, ZapA, FtsI and FtsW, again showing a direct interaction between an early (ZapA) and late (FtsN, FtsI) division proteins. No interactions were found between FtsN and FtsQ, between ZapA and FtsW, and ZapA and FtsQ. The interaction between ZapA and FtsI was not identified in BACTH^33^. In another study, bimolecular fluorescence complementation (BiFC) assays identified interactions between six divisome protein pairs; ZipA-FtsZ; ZipA-ZapA; ZapB-FtsZ; ZapB-ZapA; ZapB-ZipA and ZapB-ZapB^34^. Finally, various co-immuno precipitation (co-IP) studies confirmed or revealed interactions between divisome components. FtsQ, FtsL and FtsB were shown to form a complex before moving to the mid-cell, with mid-cell migration possibly dependent on formation of this divisomal sub-complex^35^. Interaction and sub-complex formation of FtsW and FtsI as well as interactions between FtsN, PBP1A and FtsI were identified by co-IP^36^,^37^, and GFP/GST-tagged FtsQ and FtsN also interact^38^. Clearly, many cell division proteins interact. It should be noted that all methods employ tagged versions of cell division proteins that are often (over)produced from plasmid encoded genes. Oligomeric interactions comprising multiple proteins that prove that the divisome is present as a large multi-protein machinery in the cell, have so far not been shown.

To detect whether or not a complete ‘divisome’ assembly is present in the cell, we employed clear native gel electrophoresis on mildly solubilized *E. coli* cells. Here, we describe the identification of a large 1 MDa cell division protein complex, that includes at least 7 essential division proteins; FtsZ, ZipA, FtsK, FtsQ, FtsB, FtsL, and FtsN. The complex is present in cultures of rapidly dividing cells, but not in stationary phase cultures of non-dividing cells. Slight overexpression of the *ftsQ D237N* mutation that blocks cell division^39^ prevents formation of this 1 MDa complex. In addition, in cells depleted of FtsN, the 1 MDa complex is not assembled. Combined, our findings indicate that a large protein complex containing cell division proteins indeed exists. We note that this complex is very fragile and sensitive to the expression of tagged cell division proteins. Our findings have implications for the reconstitution of the divisome *in vitro* as well as for the interpretation of data obtained with GFP-fusions to cell division proteins.

## Results

### Native Page reveals a cell division protein complex in exponentially growing cells

To study whether cell division proteins form a large multi-protein complex in the cellular envelope of *Escherichia coli*, total cell fractions of mildly solubilized bacteria were analyzed by native page. Native gels were blotted and probed with antibodies against the essential cell division protein FtsQ, as FtsQ sits in the middle of the hierarchy of divisome assembly and has been reported to interact with nearly all divisome components^12^,^39^. *E. coli* was sampled during mid exponential growth in rich medium, when most of the cells are engaged in cell division^40^. Native page of mildly solubilized cells from the exponential phase followed by Western blotting and probing with antibodies against FtsQ, revealed that most FtsQ was present in a strong band at a size of about 1 mega Dalton (MDa, Fig. 2A). Additional bands were observed around 600, 400 and 150 kDa (Fig. 2A). As a control, cells were sampled during early stationary growth, when cell division is stalled and cells lower their metabolism to a level that just allows cell maintenance^41^. The 1 MDa band was absent from stationary phase cell samples (Fig. 2A), although the amount of FtsQ in both samples was comparable as revealed by conventional SDS-PAGE/Western blotting (Fig. 2B). Coomassie staining of the native gels revealed the presence of comparable protein levels (as expected as equal levels were loaded), although bands were slightly less defined in stationary phase samples (Fig. 2A). To see whether the 1 MDa band would contain other divisome components, blots of native gels were probed with antibodies against the cell division proteins FtsK and FtsN. Again, a band was detected at 1 MDa in mid exponential phase samples, but this band was either absent (FtsK) or greatly diminished (FtsN) in stationary phase cells (Fig. 2A). The levels of both FtsK and FtsN were similar in cells sampled in both growth phases (Fig. 2B).

The presence of multiple cell division proteins in the 1 MDa band suggests that this band represents a cell division protein complex that forms in the cellular envelope during exponential growth. To ensure that the 1 MDa band is not formed by an unspecific aggregate of membrane proteins, PVDF-membranes were probed with antibodies against other membrane proteins known to form complexes that can be revealed by native page. Both SecY (component of the SecYEG translocon) and F_o_c (component of the F_1_-F_o_ ATP synthase) were present in high Mw complexes, but the antibodies against SecY and F_o_c did not cross-react with the putative divisome complex band at 1 MDa. This demonstrates that our mild solubilization method does not result in aspecific aggregation of membrane proteins in a 1 MDa band, and is capable of revealing other, known, complexes of membrane proteins. Interestingly, an earlier report on SecYEG reported a 200 kDa complex in inner membrane vesicles (IMV’s), whereas tRNA-tethered MtlA-Ribosome nascent chain complexes in the presence of IMV’s resulted in 250 and 600 kDa bands^42^ suggesting that the bands we find in exponential phase cells are representative of actively translocating SecYEG. The location of the F_o_c band corresponded to the location of the ATP-synthase complex in IMV’s identified by Blue Native Page^43^.

As most divisome components are integral membrane proteins, it would be helpful if the 1 MDa complex could be studied in an environment enriched for membrane proteins. Therefore we isolated *E. coli* IMVs and analyzed these using Native Page followed by blotting. We found that the 1 MDa band is absent from IMV’s prepared from cells irrespective of their growth phase (Fig. S1). Analysis of IMV’s by 2D-CN/SDS-PAGE on large systems as described^43^,^44^,^45^, followed by blotting, revealed that FtsQ, FtsB, FtsL could still be detected in spots that suggest FtsQLB subcomplexes, but not at 1 MDa (Fig. S2). The FtsQ/FtsB/FtsL interaction has previously been shown to be stable enough to be detected via membrane isolation and immunoprecipitation^35^. Combined, these results suggest that membrane isolation methods disrupt the 1 MDa divisome complex that we observe in whole cell samples, with some interactions still conserved in more stable sub-complexes.

### Expression of a FtsQ-mutant or depletion of FtsN disrupt cell division protein complex formation

**Figure 1:**
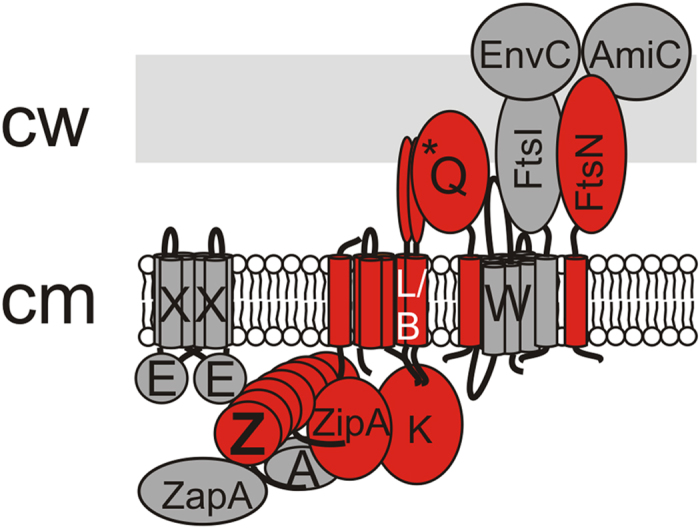
Schematic representation of *E. coli* divisome. Schematical representation of the core players in cell division FtsE/X, FtsZ, FtsA, ZipA, ZapA, FtsK, FtsQ/L/B, FtsW/I, FtsN, AmiC and EnvC situated in the cellular envelope (cm: cell wall, cw: cell wall) in a hypothetical divisome formation. Divisome components identified in the 1 MDa complex are highlighted in red. The asterisk (*) in FtsQ schematically indicates the location of D237, that is required for the interaction with downstream partners FtsL/B.

To investigate whether the 1 MDa complex found in Native PAGE is representative of the divisome, we tested whether the complex is still formed when divisome formation is blocked via introduction of excess FtsQ-D237N. The FtsQ-D237N mutant is blocked in interaction with FtsB/L (see Fig. 1) and expression of this mutant prevents the completion of the divisome complex *in vivo* as revealed by microscopy^39^. *ftsQ* (control) and *ftsQ-D237N* were expressed from a plasmid in *E. coli* in addition to the chromosomal *ftsQ*. As expected, expression of *ftsQ-D237N* resulted in cell elongation, indicative of a division defect, whereas mild overexpression of *ftsQ* had no effect (Fig. S3). Mild overexpression of *ftsQ-D237N*, but not of *ftsQ*, resulted in the almost complete disappearance of both FtsQ and FtsN from the 1 MDa protein band observed by CN-PAGE (Fig. 3A). This result strongly suggests that the 1 MDa complex is indeed the divisome, as formation of this complex is blocked by the expression of *ftsQ-D237N* as expected^39^. Also, this finding suggests that the incomplete divisome is quite unstable, as no bands at lower molecular weight could be detected in the sample where the mutant FtsQ D237N was expressed. In our experiments, FtsQ and FtsQ-D237N were only slightly (less than twofold higher) overexpressed in cultures induced with 0.2% arabinose, and levels of FtsN were close to equal in all samples (Fig. 3B). A slight excess of wild type FtsQ did not block divisome assembly (Fig. 3A), in agreement with previous findings that showed that *ftsQ* expression levels of up to fivefold higher than endogenous levels do not cause any severe effects^12^.

To further investigate whether the 1 MDa complex found in Native PAGE is representative of the divisome we depleted cells of the essential cell division protein FtsN via controlled induction/repression of *ftsN* from a plasmid in a *ftsN* chromosomal knockout strain^46^. FtsN, a late arrival at the division site^12^, is involved at stabilizing the divisome after FtsQ already joined the maturing complex^47^,^48^. Analysis of cells expressing, or depleted of FtsN, by CN-PAGE revealed that the presence of FtsQ in the 1 MDa band was dependent on the presence of FtsN. In cells depleted from FtsN, several bands that contain FtsQ can be observed, with a strong band at approximately 200 kDa, likely representative of subcomplexes containing FtsQ (Fig. 3C, compare Fig. S2). Western blotting confirmed depletion of FtsN, whereas equal levels of FtsQ were observed irrespective of the presence of FtsN (Fig. 3D).

Summarizing, the disruption of the 1 MDa protein complex by the mutant FtsQ D237N that blocks divisome assembly, as well as disruption of the complex via depletion of FtsN, indicates that this complex is indeed representative of the divisome.

### Multiple divisome proteins are present in the 1 MDa complex

The cell division complex of about 1 MDa, found in the native gel, was further characterized by band excision and analysis by conventional SDS-PAGE and Western blotting. This was done because several of the antibodies used (below) did not give sufficiently clear signals on blots of native gels. Several coomassie stained bands that could represent the 1 MDa complex were excised from a native gel, loaded on a conventional SDS-PAGE gel, and analyzed for the presence of FtsQ. One specific band from the coomassie stained clear native gel was found to contain FtsQ and thus this band matched the 1 MDa divisome complex (Fig. S4). Having identified the right band containing the divisome, the procedure was repeated and the resulting blots were probed with antibodies against specific cell division proteins. As a control, a gel fragment was excised at the same height from the lane containing the sample from early stationary phase cells. It was found that the band of interest, excised from the native gel, contains at least FtsZ, ZipA, FtsK, FtsQ, FtsL, FtsB and FtsN (Fig. 4A). In contrast, these proteins were not identified in the control fragment from the late stationary growth-phase (Fig. 4A), although in all cases the division proteins were present in comparable amounts in cells from mid-exponential and stationary growth phases, as determined by SDS-PAGE followed by Western blotting (Fig. 4B). Taken together, these observations demonstrate that the large complex of about 1 MDa contains many essential cell division proteins, spanning the range from early to late arrivals at the division site^12^.

In addition, protein identification by mass spectrometry was performed on the native gel fragment of interest containing the 1 MDa cell division complex. Over 300 proteins were identified in the native gel fragment, including FtsZ and ZipA (Supplementary table S1, S2, Supplementary file S1). However, no other cell division proteins were identified. FtsZ was identified in the native gel fragment of interest excised from the exponential phase lane as well as in the control fragment excised from the stationary phase lane; ZipA was exclusively identified in the gel fragment excised from the exponential phase lane. We cannot comment on the specificity of the presence of the proteins in these bands though—FtsZ and ZipA are present at high copy numbers in the cell^26^ and next to these proteins many other abundant proteins were present in the gel fragments, most likely the result of the application of crude, whole cell fractions to the Native PAGE. Other cell division proteins such as FtsQ have a low copy number in the cell (25–50 copies)^49^. Thus, the detection limit of identification by mass spectrometry is probably too high to detect most cell division proteins in the excised 1 MDa band fragments.

### Influence of tags to cell division protein FtsQ on complex formation

The addition of tags to cell division proteins of interest is routinely used for various purposes, e.g. purification, interaction studies or the observation of cellular localization via fluorescence microscopy. Tagging cell division proteins with GFP or other tags often results in functional proteins, and so we investigated whether tagged versions of FtsQ can also be found in the 1 MDa complex. This would also facilitate detection of components of the divisome against which no antibodies have been raised. GFP-FtsQ (N-terminal fusion of GFPmut2-tag (27 kDa)) is routinely used in fluorescence microscopy, and can rescue the temperature sensitive *E. coli* LMC531 strain^50^, that contains the chromosomal *ftsQ1* mutation (E125K) that misfolds at temperatures above 30°C. FtsQ_flag3_ (C-terminal FLAG epitope-tag, 24 residues) interacts with partner proteins FtsB/L and restores viability to an FtsQ-depletion strain in which the synthesis of endogenous FtsQ is blocked^51^. Thus, both the tagged FtsQ variants are expected to make all necessary essential interactions with other divisome partners. Both of the constructs were expressed as an extra copy from a plasmid, alongside the endogenous chromosomal *ftsQ*, and cells were sampled during mid-exponential and early stationary growth-phase and analyzed by clear native page, as described above. For the strain that expresses FtsQ_flag3_, two bands could be observed in the exponential phase sample when blotted and probed with antibodies against FtsQ; the upper band that corresponds to the original cell division complex at 1 MDa, and a more intense and broader band that runs roughly between 800 and 600 kDa (Fig. 5A). A similar pattern was found after Western blotting and probing with antibodies against the FLAG-tag (Fig. 5B). This indicates that FtsQ_flag3_ ends up in the same cell division complex as the endogenous FtsQ, but also in a different complex.

For the strain that expresses GFP-FtsQ a set of two bands is found in the exponential phase sample when GFP-FtsQ was visualized by fluorescence scanning of the native gel; a broad intense band running approximately from 800 to 700 kDa and a fainter band at 400 kDa (Fig. 5C). These bands were also found when the gel was blotted and probed with antibodies against FtsQ. This indicates that GFP-FtsQ ends up in a set of different complexes of a lower molecular mass than the 1 MDa division complex. The strong signals observed in the bands below 1 MDa in the FtsQ_flag3_ and GFP-FtsQ expressing cells suggested that the amounts of the tagged proteins in the cells were much higher than those of endogenous FtsQ, but analysis of whole cell samples by SDS-PAGE/Western blotting revealed that the levels of the tagged proteins were similar to those of endogenous proteins (Fig. 5D). So, mild expression leads to integration of FtsQ_flag3_ in the division complex, next to the presence of FtsQ_flag3_ in complexes of lower molecular weight (but still quite sizeable), whereas GFP-FtsQ seems to associate into other large complexes that are separate from the 1 MDa complex. A potential failure of GFP-tagged FtsQ to assemble into a complex was supported by overexpression of GFP-FtsQ D237N in a wild type background—overexpression of FtsQ D237N was more effective at blocking growth than overexpression of GFP-FtsQ D237N (Fig. S5, supplemental text).

## Discussion

**Figure 2:**
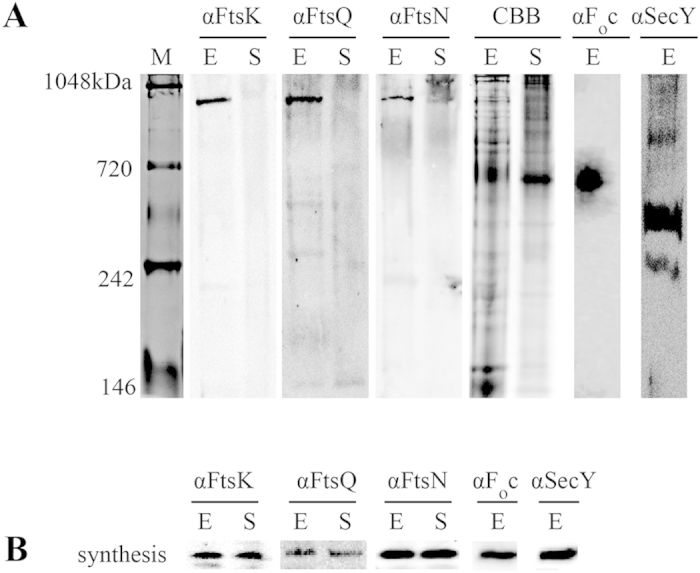
Cell division proteins are present in a 1 MDa band after native electrophoresis. (**A**) Native Page of total cell fractions of *E.coli* MC4100 sampled at mid-exponential growth phase (E) and early stationary growth phase (S), followed by Western blotting and probing with antibodies for specific cell division proteins α-FtsK, α-FtsQ, α-FtsN and control proteins α-F_o_c and α-SecY. On the far left side, a coomassie stained lane containing Native Mark^TM^ unstained protein standard (novex). In the middle, the blots with specific antibodies against cell division proteins α-FtsK, α-FtsQ, α-FtsN. Next to the right, the two coomassie stained lanes containing total cell fractions sampled at mid-exponential growth phase (E) and early stationary growth phase (S). On the far right, the blots with specific antibodies against control proteins α-F_o_c and α-SecY. The antibodies for specific cell division proteins α-FtsK, α-FtsQ, α-FtsN give the strongest signal in the mid-exponential growth phase samples at a height corresponding to a molecular weight of approximately 1 MDa, in contrast to the stationary samples where no clear signal could be detected. Striking, the signals from the antibodies against the three different cell division proteins all come from the same height. The antibodies for the control protein F_o_c (subunit of the ATP synthase machinery) give a signal at the height of roughly 500 kDa. The antibodies for the control protein SecY (SecYEG translocon) give two strong signals at the heights of circa 250 and 440 kDa. (**B**) Protein expression control. The presence and/or synthesis of cell division and control proteins was monitored in both exponential phase and stationary phase samples by SDS-Page/Western Blotting. All cell division proteins were found to be present/synthesized in equal amounts in both exponential and stationary samples.

**Figure 3:**
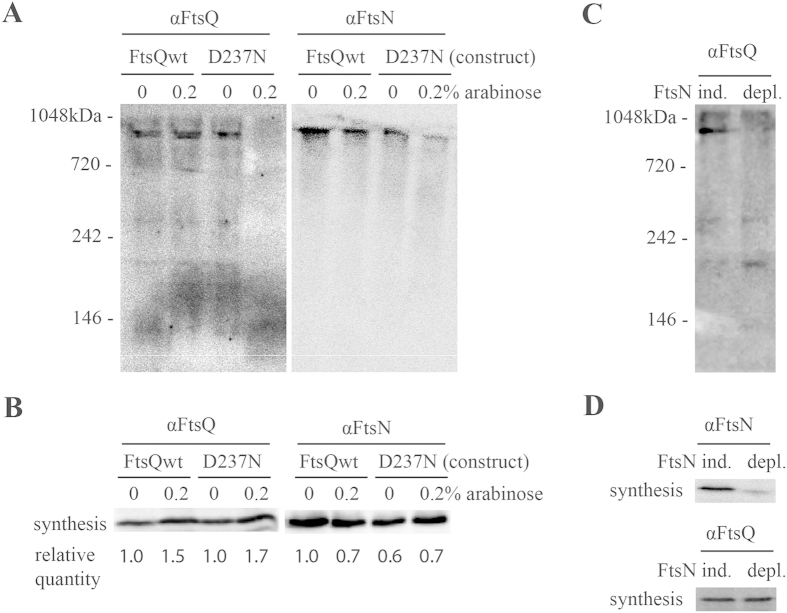
An FtsQ mutant and depletion of FtsN disrupts the 1 MDa complex. (**A**) Western blotting of Native Page of total cell fractions of *E.coli* MC4100 carrying an additional copy of either FtsQ or FtsQ D237N without or with induction with 0.2% (w/v) arabinose, followed by Western blotting and probing with antibodies for cell division proteins α-FtsQ (left) and α-FtsN (right). (**B**) Protein expression control. The presence and/or synthesis of FtsQ and FtsN was monitored in all cell samples from panel (**A**). The relative quantity of FtsQ and FtsN as detected with immunoblotting was analyzed with FIJI (ImageJ for biological sciences). The levels of FtsN remain fairly constant under all tested conditions. (**C**). Native Page of total cell fractions of FtsN-expressing (left, ind.) and depleted (right, depl.) *E. coli* JOE 565, followed by Western blotting and probing with antibodies against FtsQ. (**D**). Protein expression control. The presence of FtsN and FtsQ was monitored in all cell samples from panel (**C**).

Although the existence of the ‘divisome’ as a complex of many essential cell division proteins has been hypothesized widely and for quite some time^1^,^2^,^3^,^4^, this study is the first to identify a multi-protein complex by native PAGE that contains at least seven essential division proteins in one, 1 MDa sized, band. This is notably different from other studies in which one to one protein interactions were identified by various techniques, generally using fusion-protein constructs. We think the 1 MDa complex represents a (sub)assembly of divisome proteins involved in cell division as the presence of the complex is dependent on active cell division (Fig. 2), and the complex can be disrupted by the expression of a FtsQ mutant that blocks division, as well as via depletion of FtsN (Fig. 3). In general, an impaired ability to interact or the absence of essential cell division proteins prevents formation of the 1 MDa complex at an immature stage and/or destabilizes the complex at a more mature stage.

**Figure 4:**
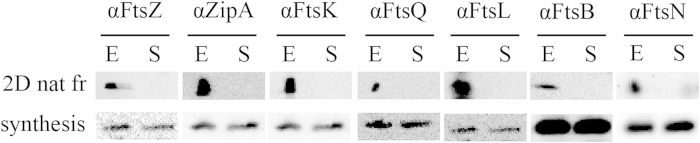
Cell division proteins present in the 1 MDa band probed by immunoblotting. A fragment (2D nat. fr.) was excised from Native Page at the molecular weight of approximately 1 MDa, where the cell division complex was found. Gel fragments of exponential growth phase samples (E) and stationary phase samples (S) were ran on SDS-Page, subsequently blotted and probed with antibodies for specific cell division proteins as indicated. For reference purposes, samples of cells sampled during exponential- and stationary growth phase were run on SDS-Page, blotted and probed in parallel to the Native Page gel fragments to investigate if the tested cell division proteins were present/expressed (synthesis).

The 1 MDa divisome complex contains proteins that localize early as well as late to the division site^12^, and contains cytosolic and integral inner membrane proteins. The 1 MDa band was only identifiable after immunodetection of various cell division proteins – and in some cases these needed to be resolved by SDS-PAGE before detection was possible (Fig. 4). A logical next step would be to analyse the native gel fragments by Mass Spectrometry. This was done but apart from FtsZ and ZipA no other cell division proteins were detected (Supplementary Table S1, S2, Supplementary file S1), most likely because of the low abundance of most division proteins, which, except for ZipA, were also never detected in other analyses of the *E. coli* membrane proteome^43^,^44^,^52^,^53^,^54^,^55^. Although most of the essential cell division proteins that have been identified thus far where found to be present in the complex, we cannot state that this complex contains all the proteins that are necessary for division. Also, we cannot make any conclusions about the stoichiometry of proteins in the complex. The molecular masses of the detected proteins are: FtsZ, 40.3 kDa; ZipA, 36.5 kDa; FtsK, 147 kDa; FtsQ, 31.4 kDa; FtsL, 13.6 kDa; FtsB, 11.6 kDa and FtsN, 35.8 kDa. In total, this makes 316.2 kDa – but with several proteins already known to multimerize a mass of 1 MDa can be imagined, however the presence of other proteins in this complex is also possible.

The 1 MDa division complex can only be observed under specific conditions: a population of actively dividing cells is required, and cell samples need to be minimally treated before clear native page. We find that membrane isolation methods such as sonication or French press followed by steps of (ultra)centrifugation disrupt the divisome complex, in contrast to careful solubilisation of cells. Some of the stronger subcomplexes (FtsQ/L/B, Fig. S2) can still be observed at lower molecular weight after native page of IMV preparations. Our finding of a SecY containing complex around 500 kDa (Fig. 2), which resembles the size of an activated Sec translocase^42^, also suggests that some interactions are lost upon membrane isolation. Thus, the divisome complex is formed by a loose assembly of proteins. This loose assembly might be due to the task of the divisome: after division, a quick disassembly is required to prevent another round of division, and untimely assembly of the divisome during the cell cycle should also be prevented.

These findings may have implications for *in vitro* reconstitution trials of the divisome. First, the environment in which the reconstituted divisome could form has to be taken into consideration; our data suggest that this environment should mimic the complete cellular envelope. Second, since we found no divisome complex in our stationary phase sample, conditions must be met that favor division; these conditions range from sufficient amounts of ATP, GTP, substrates for peptidoglycan-synthesis to proper membrane potential. Since cell division is a process that is directly or indirectly entangled with so many other cellular processes, *in vitro* reconstitution of the full division process is an enormous challenge and may require a setup that will closely resemble an *in vivo* system.

**Figure 5:**
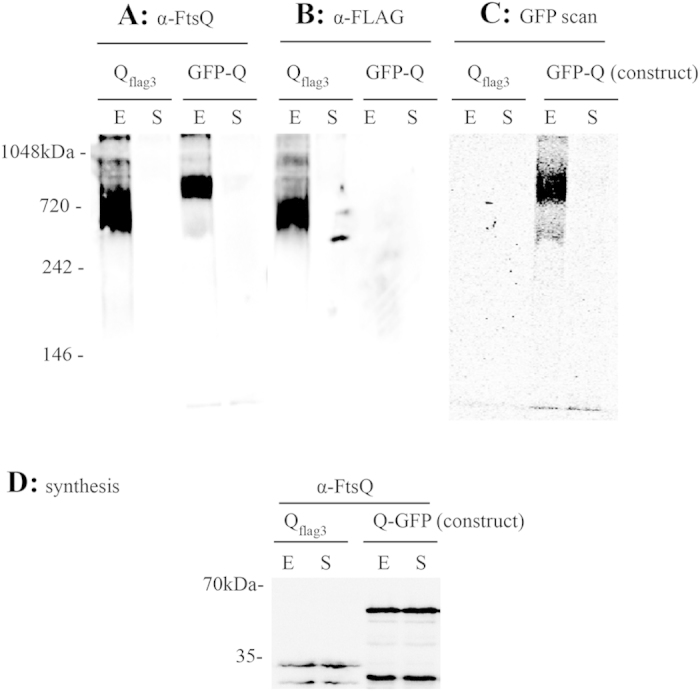
Tagged versions of FtsQ assemble into different subcomplexes. Native Page of total cell fractions expressing FtsQ_flag3_ and GFP-FtsQ sampled at mid-exponential growth phase (E) and early stationary growth phase (S), followed by in-gel fluorescence scanning for GFP (**C**) and followed by Western blotting and probing with α-FtsQ antibodies (**A**) or α-FLAG antibodies (**B**). (**D**) Protein expression control. The presence of FtsQ, FtsQ_flag3_ and GFP-FtsQ was monitored in the cell samples from panels (**A–C**).

Finally, we would like to note the effect of protein tags on the presence of FtsQ in the divisome. A widely used N-terminal GFP-fusion to FtsQ, that is functional in complementation assays and that has been used to assess the effects of FtsQ mutations on localization of FtsQ itself and other proteins^39^, is predominantly present in other complexes than the one at 1 MDa (Fig. 5). The GFP-fusion may induce protein clustering as described for oligomerizing proteins^56^, or it may hinder the interaction of non-essential cell division proteins with the complex, resulting in the formation of complexes at lower molecular masses than found with endogenous FtsQ. Also, a small 3xFLAG tag fused to the C-terminus of FtsQ, again resulting in a fully functional version of FtsQ, is present at 1 MDa but also in other complexes (Fig. 5). Nearly all studies on protein-protein interactions at the division site so far have been performed with tagged versions of these division proteins (often two proteins are tagged simultaneously), expressed from ectopic locations. Thus, care has to be taken to make sure that these proteins behave as the wild type proteins, as the extra complexes observed may be indicative of interactions with proteins that do not occur in a wild type situation. This resembles findings of Robichon *et al.* (J. Bact 2008)^57^ who noted that not all interactions found using the Bacterial Two Hybrid approach can be reproduced using other methods. Our identification of the 1 MDa native divisome complex and the methods described in this paper may serve as control experiments to further test the impact of tags on the assembly of cell division proteins into the divisome.

## Materials and Methods

### General

Bacterial strains and plasmids used are listed in Table 1.

### Sample preparation and native page

Samples: Overnight cultures of *E. coli* MC 4100 were diluted to an optical density (600 nm, OD_600_) of 0.05 and grown in Lysogeny Broth Lennox (LB) with shaking at 37 °C until OD_600_ of 0.3–0.4 (mid exponential growth phase) or 3.5–4.0 (early stationary phase). Overnight cultures of *E. coli* MC 4100 carrying pBAD24 FtsQ wt or pBAD24 FtsQ-D237N were diluted to an OD_600_ of 0.05 and grown in LB containing respectively chloramphenicol (25 μg/ml) or ampicillin (100 μg/ml ) with shaking at 37 °C until OD_600_ of 0.2. Then, the cells were diluted 1:1 in LB containing 0.4% (w/v) arabinose (final concentration 0.2%) to induce the extra *ftsQ* wt or *ftsQ-D237N* and cells were allowed to grow for 2 mass doublings until an OD_600_ of 0.4–0.5.

Overnight cultures of *E. coli* MC 4100 carrying pTHV trc GFP-FtsQ wt were diluted to OD_600_ 0.05 and grown in LB containing ampicillin (100 μg/ml ) with shaking at 37 °C until the optical density of 0.3–0.4 (mid-exponential) and 3.5–4.0 (early stationary phase) which allowed the expression of *gfp-ftsQ* without the presence of inducer (isopropyl-thiogalactopyranoside, IPTG) from a leaky *trc* promoter. ON cultures of *E. coli* CBR310 were diluted to an OD_600_ of 0.05 and grown in LB containing kanamycin (25 μg/ml), chloramphenicol (10 μg/ml), ampicillin (25 μg/ml), 0.2% (w/v) L-arabinose and 0.1 mM IPTG with shaking at 37 °C until OD_600_ of 0.3–0.4 (mid exponential phase) and 3.5–4.0 (early stationary phase) which allowed the expression of ftsQ_flag3_ at endogenous levels.

Overnight cultures of E. coli JOE 565 were diluted 1:250 in Lysogeny Broth Lennox (LB) containing chloramphenicol (10 μg/ml) and 0.02% arabinose or 0.02% glucose and grown with shaking at 37 °C until OD_600_ of 0.3–0.4 (mid-exponential growth phase).

Cells were harvested by centrifugation in an Eppendorf 5424 tabletop centrifuge at max speed for 1.5 min at room temperature (RT); from this step on samples were cooled; either on ice or at 4 °C. Cell pellets were processed immediately by resuspension of 1.5 optical density units (ODU’s) of cell material in 90 μL ice cold resuspension buffer (750 mM aminocaproic acid; 50 mM Bis Tris; pH 7.0) containing lysozyme (2.5 mg/ml) to digest cell walls and put on ice for 15 minutes. After lysozyme treatment, 10 μL of dodecyl-maltoside (DDM, 10% w/v) was added to the cell suspensions to solubilize cell membranes and the suspensions were put on ice for another 15 minutes. Subsequently, the suspensions were centrifuged (tabletop; 1 minute; max speed) to remove debris and non-solubilized cell material and 90 μL of supernatant was added to 10 μL concentrated (10x) ponceau loading buffer (0.1% ponceau S V/V, 50% glycerol V/V) for clear native page (CN-PAGE). To monitor the solubilization procedure, protein- as well as DNA concentrations in prepared samples were measured with the nanodrop ND-1000 photospectrometer at wavelengths of 280, 260 and 230 nanometer respectively. In exponential phase as well as stationary phase samples comparable protein- and DNA concentrations were measured of on average 2.5 μg/μl and 350.0 ng/μl respectively. Thus, exponential phase cells solubilize to the same extent as stationary phase cells during the sample preparation as described above. Subsequently, the samples were loaded onto a native gel; in each slot, 25 μg of protein was loaded. Alongside the samples, a Native Mark^TM^ unstained protein standard (Novex, Life Technologies) was loaded. Native gels were casted in Biorad mini protean cell systems (0.75 mm thick) as described^58^, with the exception that the separation layer of the native gels contained 10% duracrylamide (w/v) throughout. CN-PAGE was performed as described^58^ with electrophoresis at 80 V for 1 hour and 150 V for 4 hours at 4 °C. After electrophoresis, gels were either stained with coomassie brilliant blue (R-250, CBBr), scanned for fluorescence or processed for Western blotting.

### Western blotting and Fluorescence scanning of native gels

Native gels were scanned with a Typhoon Trio (GE Healthcare) scanner in fluorescence acquisition mode; excitation: 488 nm, emission: 526 nm.

Western blotting was done as follows: first, the native gels are pretreated by immersion in a solution containing 1% (w/v) dithiothreitol (DTT) and 2% (w/v) sodium-dodecylsulfate (SDS) at RT for 15 minutes. The treatment breaks the in-gel protein complexes and facilitates the transfer of proteins from the gel to the PVDF-membrane, which was subsequently done by semi-dry Western blotting. The blots were probed with antibodies against a series of cell division proteins α-FtsQ, α-FtsK, α-FtsN (kind gifts from the De Gier, Weiss, Beckwith, Vicente and Den Blaauwen labs), α-FLAG (Sigma-Aldrich) and control proteins (α-SecY, α-F_o_c: own collection). Subsequently, immunodetection was done using alkaline phosphatase conjugated anti-rabbit IgG, F(ab’)_2_fragment (Sigma) and CDP-star (Roche). Chemiluminescence was detected using a Fuji LAS-4000 imager. Whole cell samples from the same material were run on SDS-PAGE gels, Western blotted, and probed to compare total protein levels in the cell samples. In the case of FtsQ wt and FtsQ D237N overexpression, the relative quantity of the probed proteins (thickness of bands) was analyzed with FIJI^59^.

### Two dimensional protein electrophoresis of the cell division protein complex

Bands from a CBBr stained native gel that could correspond to the observed 1 MDa complex were cut from the native gel, transferred to a solution containing 1% DTT (w/v) and 2% SDS (w/v) at room temperature fot 15 minutes, followed by immersion into a solution containing 2% SDS (w/v) and 260 mM iodoacetamide^60^,^61^. After this treatment, the pieces of gel were pasted into the slots of a 10% SDS-duracrylamide (w/v) gel that were cast in Biorad mini protean cell systems (1.0 mm thick). The gel fragments were sealed in the slots with a preheated solution containing 1% (w/v) low gelling agarose and 0.5% SDS (w/v) as described^45^. For reference purposes, whole cell samples were prepared in SDS-Page sample buffer, heated at 90 °C for 5 minutes and subsequently pipetted onto and absorbed into small pieces of Whatman filter paper. These references were pasted into slots next to the pieces of native gel as well as a Pageruler^TM^ prestained protein ladder (Thermo Scientific). After loading, the gel was kept at room temperature for 1 hour allowing the low gelling agarose/SDS solution to cool down and solidify. Then, protein electrophoresis was performed in two steps of 50 V for 1 h and 120 V for 3 h, followed by Western blotting. Initially, the blot was probed with α-FtsQ antibodies to identify which Coomassie stained band corresponded to the 1 MDa complex. Subsequently, other native gels were run with samples from mid exponential and early stationary phase and the identified divisome band was cut from the lane with the exponential phase cells and a fragment at identical height was cut from the lane containing the stationary phase sample. These bands were treated as described above and run on 10% SDS-duracrylamide gels, Western blotted and the blots were probed with antibodies against a series of cell division proteins: α-FtsB, α-FtsL, α-FtsQ, α-FtsK, α-FtsN, α-FtsZ, α-ZipA (kind gifts from the De Gier, Beckwith, Vicente and Den Blaauwen labs), as described above.

### Mass spectrometry

Mass spectrometry for protein identification was essentially performed as described^62^. The supplementary file S1 containing the raw data of the experiment can be viewed with Scaffold proteome software (http://www.proteomesoftware.com/products/scaffold/).

### Microscopy

Cells were fixed by incubation in a 2.8% formaldehyde (v/v)/0.04% glutaraldehyde (v/v) solution at room temperature (RT) for 60 min, at the same time that samples were taken for protein electrophoresis. After fixation, cells were washed twice in PBS and resuspended in 30 μL of PBS before mounting on agarose pads for microscopy analysis. Cells were imaged using a Nikon Ti-E microscope (Nikon Instruments, Tokyo, Japan) equipped with an Hamamatsu Ocra Flash 4.0 camera. Image analysis was performed using the software packages Fiji and the ObjectJ plugin (https://sils.fnwi.uva.nl/bcb/objectj/) and Adobe Photoshop (Adobe Systems Inc., San Jose, CA, USA).

## Additional Information

**How to cite this article**: Trip, E. N. and Scheffers, D.-J. A 1 MDa protein complex containing critical components of the *Escherichia coli* divisome. *Sci. Rep.* **5**, 18190; doi: 10.1038/srep18190 (2015).

## Supplementary Material

Supplementary Information

Supplementary Information

## Figures and Tables

**Table 1:** Relevant plasmids and bacterial strains used in this study.

Strain or plasmid	Relevant characteristics	Source or reference
***E. coli***		
MC-4100	F^–^ *araD139 ΔlacU169 relA1 rpsL150 thi mot flb5301 deoC7 ptsF25 rbsR*	Laboratory collection
CR310	JOE309 *ftsQE14::kan Δ(λattL-lom)::bla lacI*^*q*^ *pCR45/pJC10*	63
JOE 565	JOE 309 *ftsN::kan*/pJC83	46
**Plasmids**		
pJC10	pBAD33-*ftsQ*	51
pTHV032	pBAD18-*ftsQD237N*	39
pTHV039	pTrc99A_down_-*gfp-ftsQ*	12
pTHV058	pTrc99A_down_-*gfp-ftsQD237N*	39
pJC83	pBAD33-*ftsN*	46
