# Supplementary material for: A 1 MDa protein complex containing critical components of the Escherichia coli divisome
Source: Sci Rep. 2015 Dec 8;5:18190. doi: 10.1038/srep18190 (PMC4672292; doi:10.1038/srep18190)
Supplement: Supplementary Information [file srep18190-s1.pdf]

Supplemental material for:

A 1 MDa protein complex containing critical components of the Escherichia coli divisome.

Erik N. Trip and Dirk-Jan Scheffers

contents:

supplemental figures (S1-S5) with legends, supplemental tables (S1, S2), supplemental text, supplemental file S1.

Figure S1

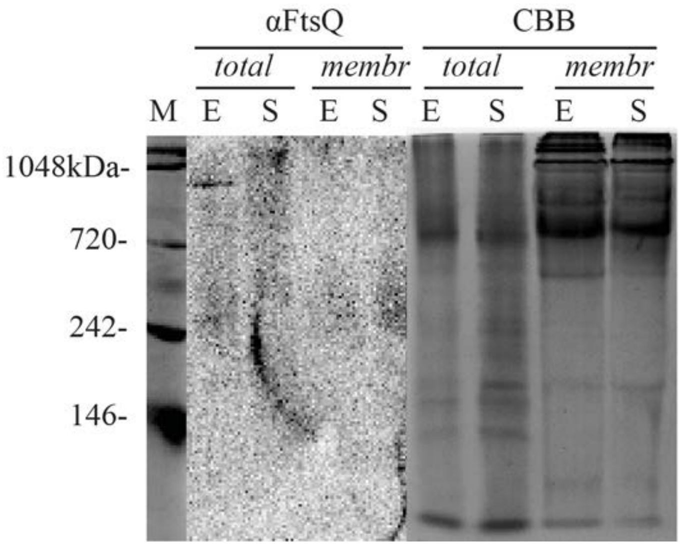

Figure S1. Native PAGE followed by Western blotting with antibodies against FtsQ of total cell fractions vs membrane fractions. On the far left side, a coomassie stained lane containing Native Mark™ unstained protein standard (novex). On the far right side, four coomassie stained lanes containing total cell fractions and membrane fractions sampled at mid-exponential growth phase (E) and early stationary growth phase (S). In the middle, an immunoblot probed with antibodies against FtsQ. Only in the total cell fraction sample taken during exponential growth the 1 MDa cell division complex is visible. In contrast to the total cell fraction sampled during stationary phase and in contrast to both membrane fractions where no bands were visible.

Fig. S2

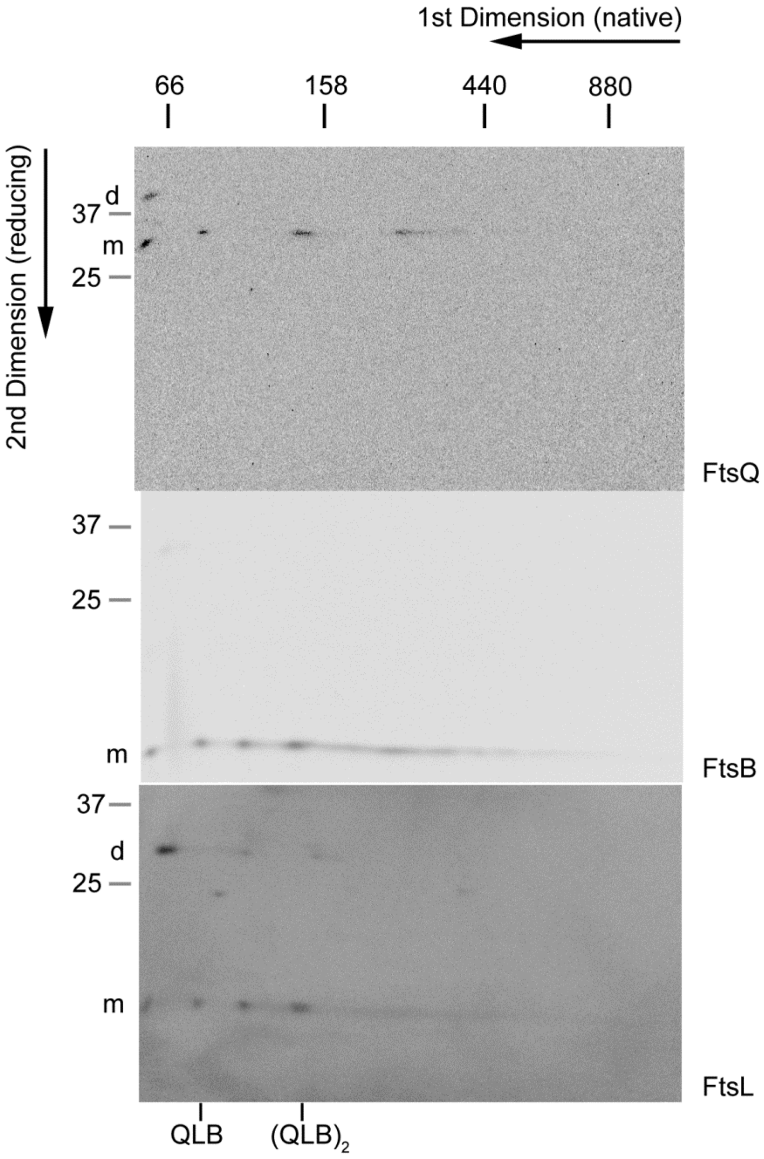

Figure S2. Two-dimensional native/SDS-PAGE of *E. coli* inner membrane vesicles (IMVs) shows complexes of FtsQ, FtsB and FtsL. Immunoblots of two-dimensional Blue Native/SDS PAGE gels reveal monomers (m) as well as dimers (d) that have not disintegrated upon equilibration in SDS-buffer. Two distinct sets of spots of FtsQ/B/L running at the same height in the 1st dimension can be resolved that could correspond to a FtsQLB complex (QLB) and a dimer of this complex (QLB_2). Additional spots corresponding to a potential complex containing FtsB and FtsL are found between the two putative QLB complexes, as well as a smeared signal at higher molecular weight, indicating a disintegrating high Mw complex.

Molecular weight markers are indicated on the sides of the blots. Two-dimensional Native/SDS-PAGE was performed as described in [64]. Briefly, *E. coli* MC4100 cytoplasmic membranes were resuspended in 750 mM Aminocaproic acid; 50 mM Bis-Tris/HCl pH 7.0, and solubilized with 0.5% dodecyl-maltoside. After removal of unsolubilized material, 0.5% Serva-Blue G was added to the samples. Samples (30 µg total protein per lane) were run on a 5-14% gradient gel cast on a GelBond PAG backing (Cambrex). Blue Native electrophoresis was performed using a step gradient with constant cooling. Ferritin (440 and 880 kDa), aldolase (158 kDa), and albumin (66 kDa) (GE Healthcare) were used as molecular mass markers. Lanes were cut from the gel, equilibrated for 15 min in 2% SDS, 5 mM tributylphosphine and 15 min in 2% SDS, 260 mM iodoacetamide, and mounted on top of a 1.5-mm-thick second dimension gel. Next to the lane, two pieces of filterpaper spotted with IMVs solubilized in SDS-sample buffer or with a prestained molecular weight marker, respectively, were loaded onto the gel. Samples were separated in the second dimension on 10% Duracryl (Genomic Solutions) gels using a Tricine-SDS buffer system in an Ettan DALTwelve system (GE Healthcare) with constant cooling. Proteins were transferred from the gels onto PVDF sheets in wet-blot systems (BioRad TransBlot, 15V, O/N, 4°C). Immunodetection was done as described in the text.

Fig. S3

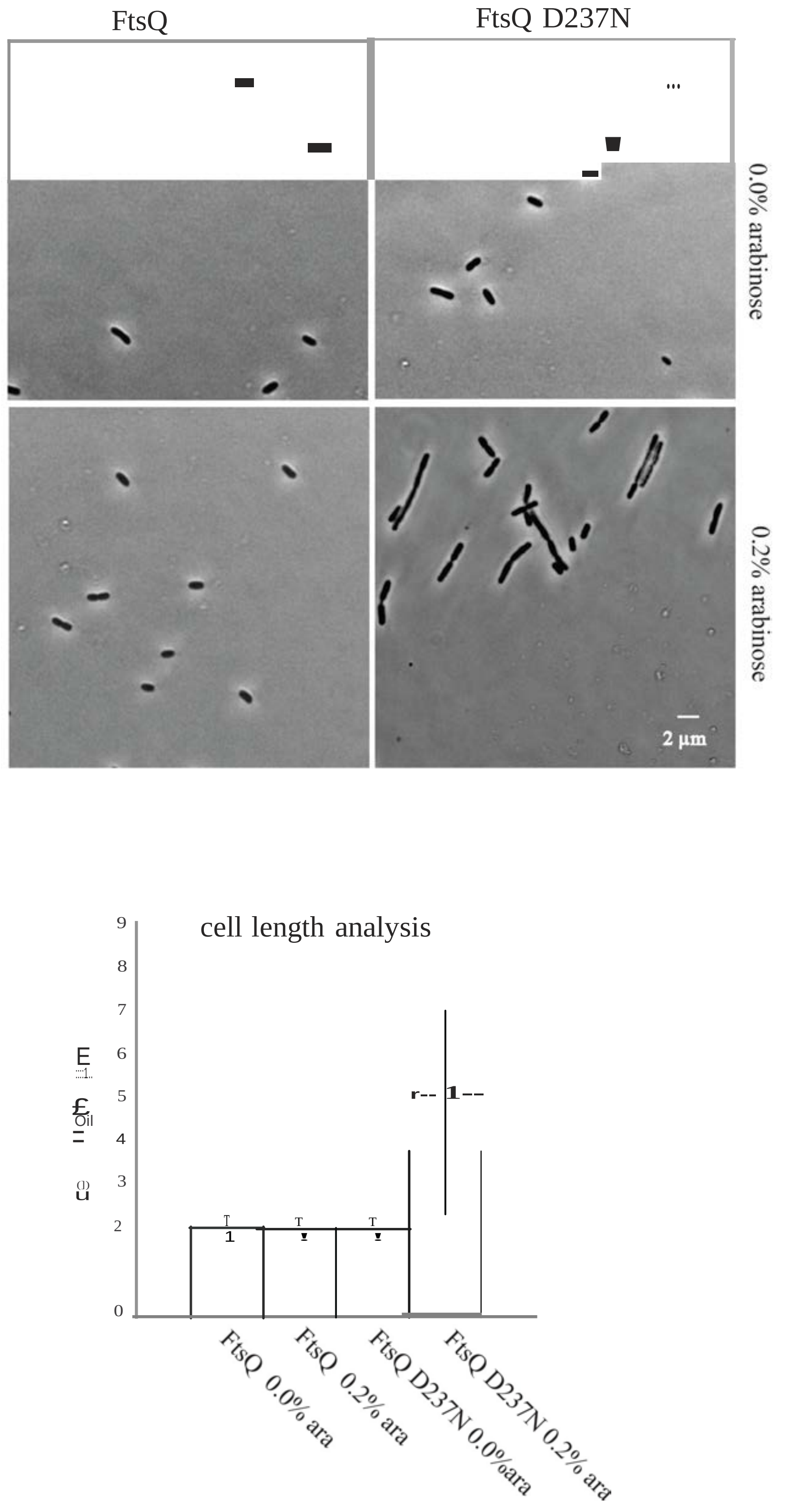
FtsQ
FtsQ D237N
0.0% arabinose
0.2% arabinose
2 µm
cell length analysis
0
2
3
4
5
6
7
8
9
FtsQ 0.0% ara
FtsQ 0.2% ara
FtsQ D237N 0.0%ara
FtsQ D237N 0.2% ara

Figure S3. Cell-length analysis of cells expressing mutant FtsQ D237N. **(A).** Phase contrast microscopy images of the cells expressing additional FtsQ or FtsQ D237N. An aliquot of cells was fixed simultaneous to sample preparation for Native Page. **(B).** Cell length analysis of the cells depicted in (A). Microscopy images were analyzed with the ObjectJ plugin for Fiji (ImageJ). Average lengths and standard deviations were: MC4100 pBAD-*ftsQ* - 0% arabinose: 2.00 ± 0.28 µm (n=173); 0.2% arabinose: 1.98 ± 0.23 µm (n=176). MC4100 pBAD-*ftsQD237N* - 0% arabinose: 1.98 ± 0.24 µm (n=135); 0.2% arabinose: 5.09 ± 2.75 µm (n=187).

Fig. S4

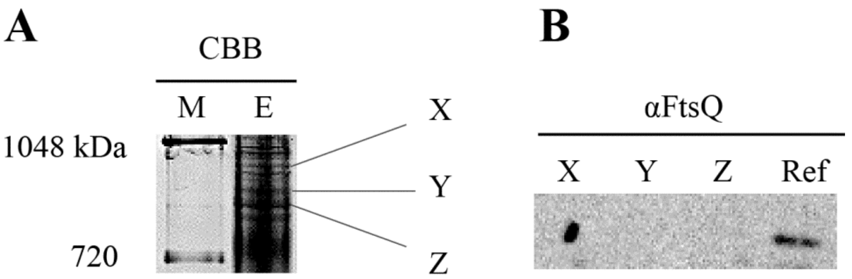

Figure S4. Pinpointing the 1 MDa band of interest in a coomassie stained native gel. A: Boxed region of the coomassie stained native gel in the region ranging from 1048 to 720 kDa. (M) is the lane containing the Native Mark™ unstained protein standard (novex), (E) is the lane containing the exponential phase sample; X, Y and Z are the bands in this lane that potentially could be the 1 MDa band of interest. **B:** these bands were excised from the coomassie stained native gel and subjected to SDS-Page and Western blotting and probed with antibodies against FtsQ as described above. (Ref) is a conventional whole cell SDS-Page sample for reference purposes. Band X was found to be the 1 MDa band of interest in the coomassie stained native gel. Subsequently, this band X from the exponential phase sample lane as well as a corresponding band from the stationary phase sample lane (control) were excised for further analysis.

Fig. S5

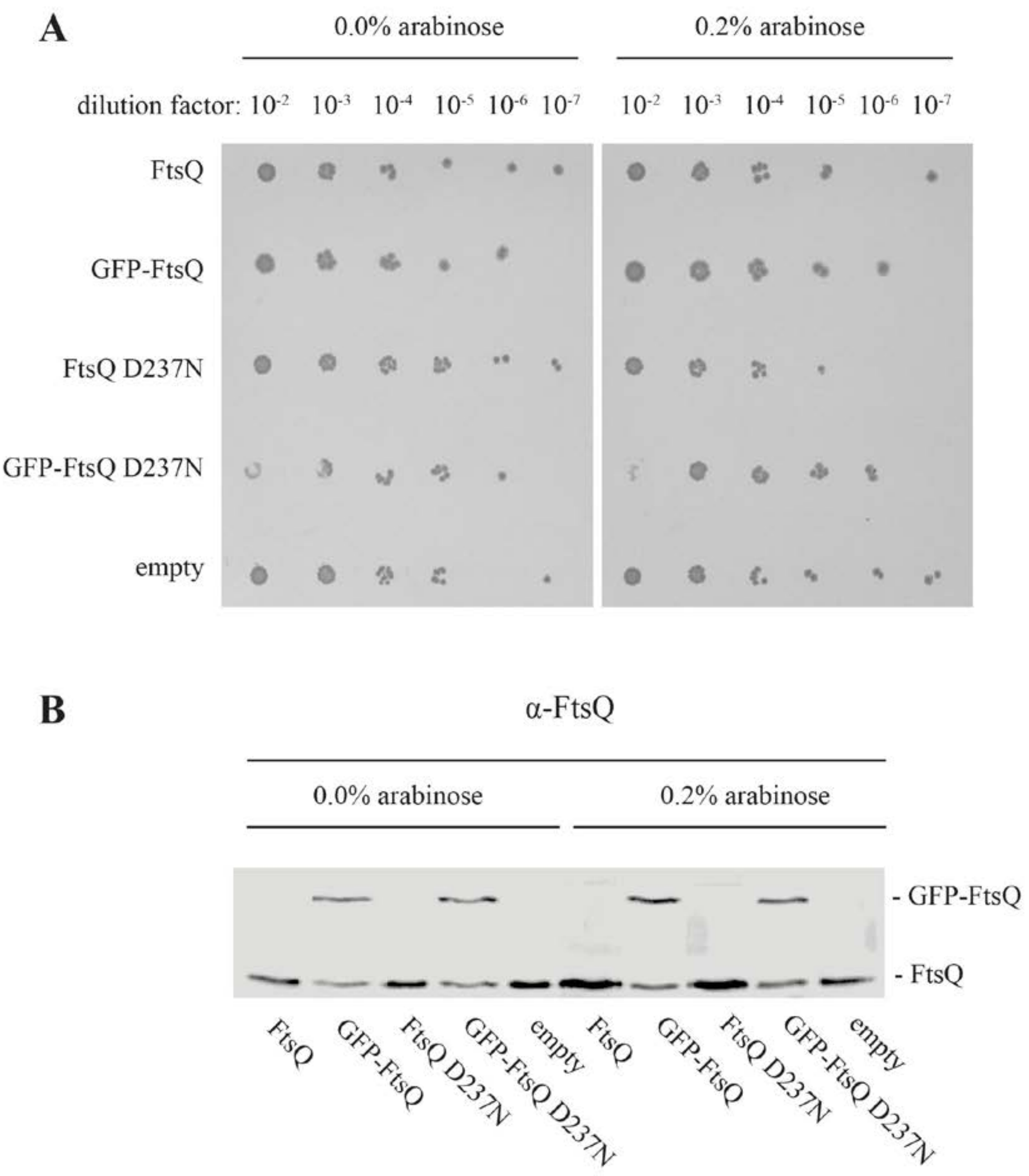

Figure S5. Influence of GFP-tags to FtsQ on cell growth. (A.) Dilution series of cells expressing additional FtsQ, GFP-FtsQ, FtsQ D237N and GFP-FtsQ D237N spotted on LB agar plates. **(B.)** Protein expression control. The presence of FtsQ and GFP-FtsQ was monitored in the cell samples from panel A.

Table S1. Proteins identified by Mass spectroscopy in the Native gel fragment of interest excized from exponential phase lane

Protein name	Protein accession numbers	Protein molecular weight (Da)	Protein identification probability	Exclusive unique peptide count	Exclusive unique spectrum count	Total spectrum count	Percentage of total spectra	Percentage sequence coverage
Serine transporter.	sp|P0AAD6|SDAC_ECOLI	46.909,00	100,00%	3	5	7	0,13%	7,69%
Inosine-5'-monophosphate dehydrogenase (	sp|P0ADG7|IMDH_ECOLI	52.022,20	100,00%	8	9	9	0,17%	23,60%
Maltose/maltodextrin import ATP-binding	sp|P68187|MALK_ECOLI	40.989,90	100,00%	5	5	5	0,09%	13,70%
Chaperone protein dnaK (Heat shock prote	sp|P0A6Y8|DNAK_ECOLI	68.984,90	100,00%	23	30	47	0,88%	39,40%
Mannose permease IID component (PTS syst	sp|P69805|PTND_ECOLI	31.304,00	100,00%	5	6	7	0,13%	21,00%
Hypothetical protein yqjD.	sp|P64581|YQJD_ECOLI	11.051,70	96,10%	1	1	1	0,02%	15,80%
D-ribose-binding periplasmic protein pre	sp|P02925|RBSB_ECOLI	30.950,50	100,00%	11	12	14	0,26%	50,30%
Dihydrolipoyl dehydrogenase (EC 1.8.1.4)	sp|P0A9P0|DLDH_ECOLI	50.557,70	100,00%	9	11	12	0,22%	23,70%
Rod shape-determining protein mreC.	sp|P16926|MREC_ECOLI	39.530,60	100,00%	3	3	3	0,06%	13,10%
Iron(III) dicitrate transport protein fe	sp|P13036|FECA_ECOLI	85.323,80	100,00%	7	7	8	0,15%	11,80%
Hypothetical protein yggE.	sp|P0ADS6|YGGE_ECOLI	26.635,50	100,00%	2	2	2	0,04%	6,91%
PTS system maltose- and glucose-specific	sp|P19642|PTOCB_ECOLI	56.628,40	100,00%	2	2	2	0,04%	4,15%
Thiamine biosynthesis protein thiI.	sp|P77718|THII_ECOLI	54.973,70	100,00%	3	3	3	0,06%	6,43%
NADH-quinone oxidoreductase chain G (EC	sp|P33602|NUOG_ECOLI	100.168,30	100,00%	2	2	2	0,04%	2,43%
Glutamate synthase [NADPH] large chain p	sp|P09831|GLTB_ECOLI	166.713,70	100,00%	8	8	8	0,15%	6,33%
UPF0234 protein yajQ.	sp|P0A8E7|YAJQ_ECOLI	19.047,00	100,00%	4	5	5	0,09%	31,40%
Hypothetical UPF0135 protein ybgI.	sp|P0AFP6|YBGI_ECOLI	26.892,70	96,10%	1	1	1	0,02%	6,88%
Protein dcrB precursor.	sp|P0AEE1|DCRB_ECOLI	19.787,70	100,00%	3	4	4	0,07%	22,70%
Dihydrolipoyllysine-residue acetyltransf	sp|P06959|ODP2_ECOLI	65.964,70	99,80%	1	1	1	0,02%	2,23%
Hypothetical protein yefG (GalF transfer	sp|P37749|YEFG_ECOLI	37.759,10	100,00%	2	2	2	0,04%	6,36%
Probable ornithine aminotransferase (EC	sp|P42588|OAT_ECOLI	46.401,60	95,20%	1	1	1	0,02%	2,80%
Succinyl-CoA synthetase beta chain (EC 6	sp|P0A836|SUCC_ECOLI	41.392,70	100,00%	4	5	5	0,09%	14,70%
DNA-directed RNA polymerase beta' chain	sp|P0A8T7|RPOC_ECOLI	155.164,10	100,00%	6	6	7	0,13%	5,83%
50S ribosomal protein L22.	sp|P61175|RL22_ECOLI	12.226,60	96,10%	1	1	1	0,02%	11,80%
Hypothetical protein yhaM.	sp|P42626|YHAM_ECOLI	45.361,20	96,10%	1	1	1	0,02%	2,52%
Serine hydroxymethyltransferase (EC 2.1.	sp|P0A825|GLYA_ECOLI	45.316,70	100,00%	2	2	2	0,04%	4,56%
DNA-directed RNA polymerase beta chain (	sp|P0A8V2|RPOB_ECOLI	150.635,90	100,00%	27	34	43	0,80%	23,40%

Pyrroline-5-carboxylate reductase (EC 1.	sp|P0A9L8|PROC_ECOLI	28.145,00	100,00%	7	11	21	0,39%	34,90%
Inner membrane protein yjiY.	sp|P39396|YJIY_ECOLI	77.860,40	100,00%	2	2	2	0,04%	3,74%
keratin 1, type II, cytoskeletal - human	KRHU2	65.495,20	100,00%	3	3	3	0,06%	5,29%
Tripeptide permease tppB.	sp|P77304|TPPB_ECOLI	53.993,30	97,80%	1	1	1	0,02%	2,60%
Maltoporin precursor (Maltose-inducible	sp|P02943|LAMB_ECOLI	49.912,80	100,00%	4	4	4	0,07%	13,00%
Elongation factor Ts (EF-Ts).	sp|P0A6P1|EFTS_ECOLI	30.291,60	100,00%	12	15	16	0,30%	40,80%
Phosphate-binding periplasmic protein pr	sp|P0AG82|PSTS_ECOLI	37.024,70	100,00%	11	11	16	0,30%	41,00%
Heat shock protein hslJ.	sp|P52644|HSLJ_ECOLI	15.166,00	100,00%	2	2	2	0,04%	19,30%
ATP synthase gamma chain (EC 3.6.3.14) (	sp|P0ABA6|ATPG_ECOLI	31.577,90	100,00%	5	5	5	0,09%	15,70%
Lipoprotein 34 precursor.	sp|P0A903|NLPB_ECOLI	36.842,30	100,00%	9	10	13	0,24%	35,20%
Protease VII precursor (EC 3.4.23.-) (Om	sp|P09169|OMPT_ECOLI	35.563,90	100,00%	10	12	20	0,37%	34,40%
Cytochrome o ubiquinol oxidase subunit I	sp|P0ABJ3|CYOC_ECOLI	22.623,20	96,10%	1	1	1	0,02%	5,39%
2,3,4,5-tetrahydropyridine-2,6-dicarboxy	sp|P0A9D8|DAPD_ECOLI	29.891,60	100,00%	3	3	3	0,06%	13,10%
Hypothetical protein yjeI precursor.	sp|P0AF70|YJEI_ECOLI	11.958,30	99,90%	1	1	1	0,02%	16,20%
UPF0283 membrane protein ycjF.	sp|P0A8R7|YCJF_ECOLI	39.392,90	99,90%	1	1	1	0,02%	3,68%
Cell division protein ftsZ.	sp|P0A9A6|FTSZ_ECOLI	40.324,00	100,00%	3	3	3	0,06%	9,14%
Tagatose-1,6-bisphosphate aldolase gatY	sp|P37192|GATY_ECOLI	30.811,50	100,00%	3	3	3	0,06%	9,86%
GTP-binding protein lepA.	sp|P60785|LEPA_ECOLI	66.571,70	99,60%	1	1	1	0,02%	1,84%
Probable ABC transporter ATP-binding pro	sp|P0A9V1|YHBG_ECOLI	26.670,30	100,00%	4	5	7	0,13%	19,60%
Purine nucleoside phosphorylase deoD-typ	sp|P0ABP8|DEOD_ECOLI	25.819,30	100,00%	2	2	2	0,04%	9,66%
Isoaspartyl dipeptidase (EC 3.4.19.-).	sp|P39377|IADA_ECOLI	41.084,40	100,00%	4	4	5	0,09%	11,50%
Thymidine phosphorylase (EC 2.4.2.4) (Td	sp|P07650|TYPH_ECOLI	47.208,30	98,20%	1	1	1	0,02%	1,59%
DNA-directed RNA polymerase alpha chain	sp|P0A7Z4|RPOA_ECOLI	36.512,10	100,00%	5	6	6	0,11%	16,70%
Galactoside transport system permease pr	sp|P23200|MGLC_ECOLI	35.551,90	96,10%	1	1	1	0,02%	3,87%
Signal peptidase I (EC 3.4.21.89) (SPase	sp|P00803|LEP_ECOLI	35.961,20	100,00%	2	2	2	0,04%	7,41%
Pyruvate kinase II (EC 2.7.1.40) (PK-2).	sp|P21599|KPYK2_ECOLI	51.226,60	100,00%	8	9	9	0,17%	19,00%
Putative xanthine dehydrogenase yagR, mo	sp|P77489|YAGR_ECOLI	78.089,00	99,40%	1	1	1	0,02%	1,23%
ATP synthase C chain (EC 3.6.3.14) (Lipi	sp|P68699|ATPL_ECOLI	8.256,70	99,70%	2	2	6	0,11%	20,30%
Phosphoenolpyruvate carboxylase (EC 4.1.	sp|P00864|CAPP_ECOLI	99.066,00	100,00%	18	18	22	0,41%	21,20%
Biotin carboxylase (EC 6.3.4.14) (A subu	sp|P24182|ACCC_ECOLI	49.321,90	100,00%	9	9	13	0,24%	20,50%
10 kDa chaperonin (Protein Cpn10) (groES	sp|P0A6F9|CH10_ECOLI	10.387,00	100,00%	4	5	5	0,09%	52,60%
Hypothetical adenine-specific methylase	sp|P39199|YFCB_ECOLI	35.002,40	100,00%	4	4	4	0,07%	13,20%

30S ribosomal protein S20.	sp|P0A7U7|RS20_ECOLI	9.553,70	100,00%	2	2	2	0,04%	23,30%
30S ribosomal protein S3.	sp|P0A7V3|RS3_ECOLI	25.852,30	100,00%	3	3	3	0,06%	13,40%
Tryptophanase (EC 4.1.99.1) (L-tryptopha	sp|P0A853|TNAA_ECOLI	52.774,80	100,00%	17	23	43	0,80%	42,70%
Aspartyl-tRNA synthetase (EC 6.1.1.12) (	sp|P21889|SYD_ECOLI	65.915,50	100,00%	6	6	6	0,11%	11,20%
Formate acetyltransferase 1 (EC 2.3.1.54	sp|P09373|PFLB_ECOLI	85.229,10	100,00%	20	34	44	0,82%	30,60%
Hypothetical lipoprotein yedD precursor.	sp|P31063|YEDD_ECOLI	14.983,50	100,00%	4	5	6	0,11%	41,60%
CTP synthase (EC 6.3.4.2) (UTP--ammonia	sp|P0A7E5|PYRG_ECOLI	60.243,40	100,00%	5	5	5	0,09%	10,70%
50S ribosomal protein L5.	sp|P62399|RL5_ECOLI	20.171,50	100,00%	11	16	25	0,47%	65,70%
50S ribosomal protein L6.	sp|P0AG55|RL6_ECOLI	18.772,30	100,00%	2	2	2	0,04%	13,10%
Phenylalanyl-tRNA synthetase alpha chain	sp|P08312|SYFA_ECOLI	36.832,50	100,00%	20	38	72	1,34%	53,80%
Protein hflK.	sp|P0ABC7|HFLK_ECOLI	45.545,50	100,00%	5	5	6	0,11%	14,80%
ATP-dependent hsl protease ATP-binding s	sp|P0A6H5|HSLU_ECOLI	49.595,20	100,00%	13	14	16	0,30%	31,40%
NADH-quinone oxidoreductase chain B (EC	sp|P0AFC7|NUOB_ECOLI	25.055,60	100,00%	2	2	2	0,04%	9,55%
Ferrienterobactin receptor precursor (En	sp|P05825|FEPA_ECOLI	82.108,40	100,00%	4	4	4	0,07%	8,98%
Putative metalloprotease yggG (EC 3.4.24	sp|P25894|YGGG_ECOLI	26.842,40	100,00%	2	2	2	0,04%	10,70%
Glyceraldehyde-3-phosphate dehydrogenase	sp|P0A9B2|G3P1_ECOLI	35.401,40	100,00%	15	20	33	0,62%	43,90%
Sulfate/thiosulfate import ATP-binding p	sp|P16676|CYSA_ECOLI	41.058,90	99,90%	1	1	1	0,02%	3,01%
Biopolymer transport exbB protein.	sp|P0ABU7|EXBB_ECOLI	26.287,80	100,00%	2	2	3	0,06%	9,84%
Protein-export membrane protein secF.	sp|P0AG93|SECF_ECOLI	35.383,50	100,00%	2	2	2	0,04%	9,91%
Lipid A export ATP-binding/permease prot	sp|P60752|MSBA_ECOLI	64.462,70	100,00%	12	13	17	0,32%	20,30%
Enoyl-[acyl-carrier-protein] reductase [	sp|P0AEK4|FABI_ECOLI	27.733,40	96,10%	1	1	1	0,02%	3,83%
Acyl-CoA thioester hydrolase ybgC precur	sp|P46130|YBHC_ECOLI	46.081,90	100,00%	7	7	10	0,19%	21,80%
Chaperone protein htpG (Heat shock prote	sp|P0A6Z3|HTPG_ECOLI	71.425,00	100,00%	16	17	22	0,41%	29,30%
Glycogen phosphorylase (EC 2.4.1.1).	sp|P0AC86|PHSG_ECOLI	93.175,30	99,60%	2	2	2	0,04%	2,21%
Alkyl hydroperoxide reductase subunit C	sp|P0AE08|AHPC_ECOLI	20.630,70	100,00%	2	2	2	0,04%	15,60%
Colicin I receptor precursor.	sp|P17315|CIRA_ECOLI	73.896,60	100,00%	11	11	12	0,22%	23,70%
Alkyl hydroperoxide reductase subunit F	sp|P35340|AHPF_ECOLI	56.178,20	100,00%	11	11	12	0,22%	26,10%
ATP synthase beta chain (EC 3.6.3.14).	sp|P0ABB4|ATPB_ECOLI	50.194,30	100,00%	19	29	52	0,97%	54,50%
Putative 6-hydroxy-D-nicotine oxidase OS=Arthrobacter nicotinovorans GN=6-HDNO PE=1 SV=1	tr|Q8GAG1|Q8GAG1_ARTNI,tr|Q8GAGL|L138H_ARTNI,tr|Q8GAGM|H130C_ARTNI	49.922,80	98,80%	1	1	1	0,02%	2,57%
Glycerol-3-phosphate transporter (G-3-P	sp|P08194|GLPT_ECOLI	50.312,50	99,60%	1	1	1	0,02%	2,43%

Chain length determinant protein (Polysa	sp|P76372|WZZB_ECOLI	36.455,60	100,00%	8	9	11	0,21%	32,20 %
PTS system N-acetylglucosamine-specific	sp|P09323|PTW3C_ECOLI	68.348,70	100,00%	2	2	2	0,04%	4,94%
Hypothetical protein yrbK.	sp|P0ADV9|YRBK_ECOLI	21.702,70	100,00%	2	2	2	0,04%	13,60 %
Probable protease htpX (EC 3.4.24.-) (He	sp|P23894|HTPX_ECOLI	31.924,10	96,10%	1	1	1	0,02%	3,75%
50S ribosomal protein L18.	sp|P0C018|RL18_ECOLI	12.769,70	100,00%	3	4	5	0,09%	22,20 %
Transketolase 1 (EC 2.2.1.1) (TK 1).	sp|P27302|TKT1_ECOLI	72.213,00	100,00%	6	6	6	0,11%	9,80%
Penicillin-binding protein 5 precursor (	sp|P0AEB2|DACA_ECOLI	44.445,50	96,10%	1	1	1	0,02%	2,48%
5-methyltetrahydropteroyltriglutamate--h	sp|P25665|METE_ECOLI	84.544,00	100,00%	12	13	16	0,30%	18,60 %
Outer membrane lipoprotein slyB precurso	sp|P0A905|SLYB_ECOLI	15.600,60	100,00%	4	8	12	0,22%	38,70 %
Formate dehydrogenase-O, major subunit (	sp|P32176|FDOG_ECOLI	112.505,50	99,90%	1	1	1	0,02%	1,67%
50S ribosomal protein L17.	sp|P0AG44|RL17_ECOLI	14.365,20	100,00%	3	4	7	0,13%	26,00 %
Pyruvate dehydrogenase E1 component (EC	sp|P0AFG8|ODP1_ECOLI	99.540,00	100,00%	32	42	62	1,16%	39,20 %
Rare lipoprotein A precursor.	sp|P10100|RLPA_ECOLI	37.527,60	100,00%	5	5	6	0,11%	16,00 %
Cystathionine gamma-synthase (EC 2.5.1.4	sp|P00935|METB_ECOLI	41.550,90	100,00%	3	3	4	0,07%	9,59%
Magnesium transporting ATPase, P-type 1	sp|P0ABB8|ATMA_ECOLI	99.468,80	100,00%	6	6	6	0,11%	8,02%
Glycyl-tRNA synthetase beta chain (EC 6.	sp|P00961|SYGB_ECOLI	76.684,10	100,00%	17	19	25	0,47%	30,80 %
Transcription elongation protein nusA (N	sp|P0AFF6|NUSA_ECOLI	54.871,40	100,00%	4	4	4	0,07%	9,09%
2-amino-3-ketobutyrate coenzyme A ligase	sp|P0AB77|KBL_ECOLI	43.117,70	100,00%	2	2	2	0,04%	5,03%
D-methionine transport ATP-binding prote	sp|P30750|METN_ECOLI	37.788,80	100,00%	5	5	6	0,11%	21,00 %
Isoleucyl-tRNA synthetase (EC 6.1.1.5) (	sp|P00956|SYI_ECOLI	104.167,10	100,00%	4	4	4	0,07%	4,27%
Lipoprotein yfgL precursor.	sp|P77774|YFGL_ECOLI	41.886,90	100,00%	6	6	7	0,13%	22,70 %
PhoH-like protein.	sp|P0A9K3|PHOL_ECOLI	40.655,80	100,00%	6	7	10	0,19%	20,10 %
Inner membrane protein yjgQ.	sp|P0ADC6|YJGQ_ECOLI	39.620,10	100,00%	2	2	3	0,06%	6,39%
Inner membrane protein oxaA.	sp|P25714|OXAA_ECOLI	61.528,00	100,00%	7	7	7	0,13%	13,10 %
EIImtl	6H-C384S-W42,6H-W42,EII-F97W,sp|P00550|PTM3C_ECOLI	67.857,70	100,00%	6	7	9	0,17%	13,00 %
30S ribosomal protein S1.	sp|P0AG67|RS1_ECOLI	61.157,40	100,00%	9	11	13	0,24%	23,00 %
Na(+)/H(+) antiporter 2 (Sodium/proton a	sp|P0AFA7|NHAB_ECOLI	56.730,50	100,00%	2	3	3	0,06%	4,87%
Elongation factor G (EF-G).	sp|P0A6M8|EFG_ECOLI	77.450,80	100,00%	18	23	35	0,65%	35,80 %
Mannose permease IIC component (PTS syst	sp|P69801|PTNC_ECOLI	27.636,40	100,00%	2	2	3	0,06%	11,70 %
RNA polymerase sigma factor rpoD (Sigma-	sp|P00579|RPOD_ECOLI	70.265,30	100,00%	4	5	5	0,09%	6,69%
30S ribosomal protein S16.	sp|P0A7T3|RS16_ECOLI	9.190,60	95,70%	1	1	1	0,02%	13,40 %

Protein-export membrane protein secD.	sp|P0AG90|SECD_ECOLI	66.634,50	100,00%	14	14	16	0,30%	28,30 %
RNA polymerase associated protein rapA (	sp|P60240|RAPA_ECOLI	109.640,50	100,00%	3	3	3	0,06%	3,21%
Preprotein translocase secY subunit.	sp|P0AGA2|SECY_ECOLI	48.514,00	100,00%	3	4	4	0,07%	8,80%
Protein ydgH precursor.	sp|P76177|YDGH_ECOLI	33.903,30	96,60%	1	1	1	0,02%	3,82%
GTP cyclohydrolase I (EC 3.5.4.16) (GTP-	sp|P0A6T5|GCH1_ECOLI	24.699,80	100,00%	5	6	7	0,13%	28,10 %
Oligopeptidase A (EC 3.4.24.70).	sp|P27298|OPDA_ECOLI	77.169,50	95,00%	1	1	1	0,02%	1,62%
Organic solvent tolerance protein precur	sp|P31554|OSTA_ECOLI	89.672,80	100,00%	13	16	20	0,37%	20,40 %
Superoxide dismutase [Mn] (EC 1.15.1.1)	sp|P00448|SODM_ECOLI	22.966,60	100,00%	5	6	11	0,21%	20,50 %
Lipoprotein bor homolog from lambdoid pr	sp|P77330|BORD_ECOLI	10.447,30	97,30%	1	1	1	0,02%	10,30 %
Cell division protein ftsY.	sp|P10121|FTSY_ECOLI	54.512,50	97,30%	1	1	1	0,02%	2,01%
DNA gyrase subunit B (EC 5.99.1.3).	sp|P0AES6|GYRB_ECOLI	89.820,80	100,00%	5	5	5	0,09%	7,85%
Keratin, type I cytoskeletal 9	K1C9_HUMAN	62.131,00	100,00%	3	3	3	0,06%	8,35%
Methionine synthase (EC 2.1.1.13) (5-met	sp|P13009|METH_ECOLI	135.868,30	100,00%	3	3	3	0,06%	3,10%
Alanyl-tRNA synthetase (EC 6.1.1.7) (Ala	sp|P00957|SYA_ECOLI	96.033,60	100,00%	35	43	60	1,12%	44,50 %
Glutamine transport ATP-binding protein	sp|P10346|GLNQ_ECOLI	26.731,30	100,00%	2	2	2	0,04%	12,90 %
Spermidine/putrescine transport system p	sp|P0AFK4|POTB_ECOLI	31.064,80	99,40%	2	2	2	0,04%	6,91%
Succinyl-CoA synthetase alpha chain (EC	sp|P0AGE9|SUCD_ECOLI	29.646,60	100,00%	6	6	7	0,13%	22,20 %
Hypothetical protein ycfM.	sp|P0AB38|YCFM_ECOLI	22.515,20	100,00%	2	2	3	0,06%	12,70 %
Adenylosuccinate lyase (EC 4.3.2.2) (Ade	sp|P0AB89|PUR8_ECOLI	51.544,10	100,00%	7	7	9	0,17%	16,40 %
Chromosome partition protein mukB (Struc	sp|P22523|MUKB_ECOLI	170.232,70	99,90%	2	2	2	0,04%	1,08%
Apolipoprotein N-acyltransferase (EC 2.3	sp|P23930|LNT_ECOLI	57.068,90	99,40%	2	2	2	0,04%	4,49%
Glucose-6-phosphate isomerase (EC 5.3.1.	sp|P0A6T1|G6PI_ECOLI	61.531,40	100,00%	3	3	3	0,06%	6,56%
Rare lipoprotein B precursor.	sp|P0ADC1|RLPB_ECOLI	21.357,30	100,00%	5	5	6	0,11%	31,60 %
L-threonine 3-dehydrogenase (EC 1.1.1.10	sp|P07913|TDH_ECOLI	37.239,60	96,10%	1	1	1	0,02%	3,23%
Hypothetical oxidoreductase yqhD (EC 1.1	sp|Q46856|YQHD_ECOLI	42.097,60	100,00%	2	2	2	0,04%	5,94%
Hypothetical lipoprotein ydcL precursor.	sp|P64451|YDCL_ECOLI	24.427,30	100,00%	4	4	4	0,07%	21,60 %
ATP-dependent Clp protease ATP-binding s	sp|P0ABH9|CLPA_ECOLI	84.208,30	100,00%	6	6	7	0,13%	9,63%
Outer membrane protein C precursor (Pori	sp|P06996|OMPC_ECOLI	40.369,00	100,00%	7	7	9	0,17%	22,60 %
50S ribosomal protein L15.	sp|P02413|RL15_ECOLI	14.981,10	100,00%	5	5	5	0,09%	39,60 %
NAD(P) transhydrogenase subunit beta (EC	sp|P0AB67|PNTB_ECOLI	48.724,00	100,00%	5	7	7	0,13%	11,50 %
Branched-chain amino acid transport syst	sp|P0AD99|BRNQ_ECOLI	46.209,90	96,10%	1	1	1	0,02%	3,19%

Tryptophanyl-tRNA synthetase (EC 6.1.1.2	sp|P00954|SYW_ECOLI	37.438,40	99,80%	2	2	2	0,04%	6,59%
ADP-L-glycero-D-manno-heptose-6-epimeras	sp|P67910|HLDD_ECOLI	34.894,30	100,00%	10	10	15	0,28%	35,50%
Hypothetical protein yiaF.	sp|P0ADK0|YIAF_ECOLI	30.158,60	100,00%	5	6	7	0,13%	24,60%
Phosphate acetyltransferase (EC 2.3.1.8)	sp|P0A9M8|PTA_ECOLI	77.041,50	100,00%	19	23	33	0,62%	31,60%
50S ribosomal protein L10 (50S ribosomal	sp|P0A7J3|RL10_ECOLI	17.580,60	100,00%	9	13	33	0,62%	51,80%
30S ribosomal protein S4.	sp|P0A7V8|RS4_ECOLI	23.338,50	100,00%	3	3	3	0,06%	13,20%
Peroxidase/catalase HPI (EC 1.11.1.6) (C	sp|P13029|CATA_ECOLI	80.025,80	100,00%	27	39	82	1,53%	39,80%
Ribose transport system permease protein	sp|P0AGI1|RBSC_ECOLI	33.452,90	96,10%	1	1	1	0,02%	4,05%
Ubiquinol oxidase polypeptide II precurs	sp|P0ABJ1|CYOA_ECOLI	34.912,30	100,00%	7	9	16	0,30%	31,10%
S-adenosylmethionine synthetase (EC 2.5.	sp|P0A817|METK_ECOLI	41.820,90	100,00%	3	3	3	0,06%	7,57%
3-oxoacyl-[acyl-carrier-protein] synthas	sp|P0A953|FABB_ECOLI	42.613,40	99,80%	1	1	1	0,02%	2,46%
Peptidyl-prolyl cis-trans isomerase D (E	sp|P0ADY1|PPID_ECOLI	68.151,40	100,00%	11	12	15	0,28%	23,80%
NADH dehydrogenase (EC 1.6.99.3).	sp|P00393|DHNA_ECOLI	47.228,80	100,00%	13	16	22	0,41%	40,40%
30S ribosomal protein S2.	sp|P0A7V0|RS2_ECOLI	26.613,10	100,00%	6	6	9	0,17%	28,30%
Hypothetical lipoprotein yifL precursor.	sp|P0ADN6|YIFL_ECOLI	7.177,40	94,70%	1	2	4	0,07%	34,30%
Putative lipoprotein yceB precursor.	sp|P0AB26|YCEB_ECOLI	20.500,30	100,00%	1	1	1	0,02%	6,45%
30S ribosomal protein S6 [Contains: 30S	sp|P02358|RS6_ECOLI	15.703,30	96,10%	1	1	1	0,02%	8,15%
UPF0304 protein yfbU.	sp|P0A8W8|YFBU_ECOLI	19.536,50	99,70%	1	1	1	0,02%	9,15%
UPF0169 lipoprotein yfiO precursor.	sp|P0AC02|YFIO_ECOLI	27.830,30	100,00%	6	6	8	0,15%	27,80%
Phenylalanyl-tRNA synthetase beta chain	sp|P07395|SYFB_ECOLI	87.378,60	100,00%	36	46	116	2,17%	47,20%
Anthranilate synthase component I (EC 4.	sp|P00895|TRPE_ECOLI	57.495,80	100,00%	8	8	8	0,15%	17,30%
D-3-phosphoglycerate dehydrogenase (EC 1	sp|P0A9T0|SERA_ECOLI	44.045,90	100,00%	9	9	11	0,21%	27,40%
Entericidin B precursor.	sp|P0ADB7|ECNB_ECOLI	4.809,50	96,10%	1	1	1	0,02%	39,60%
Uridine phosphorylase (EC 2.4.2.3) (UrdP	sp|P12758|UDP_ECOLI	27.027,50	100,00%	4	4	4	0,07%	18,30%
NAD(P) transhydrogenase subunit alpha (E	sp|P07001|PNTA_ECOLI	54.623,70	100,00%	4	4	4	0,07%	9,61%
50S ribosomal protein L25.	sp|P68919|RL25_ECOLI	10.693,60	100,00%	5	5	8	0,15%	44,70%
Nucleoside permease nupC (Nucleoside-tra	sp|P0AFF2|NUPC_ECOLI	43.477,70	100,00%	2	2	3	0,06%	5,00%
Glycerol uptake facilitator protein (Aqu	sp|P0AER0|GLPF_ECOLI	29.780,60	96,10%	1	1	1	0,02%	4,27%
Peptidoglycan-associated lipoprotein pre	sp|P0A912|PAL_ECOLI	18.824,50	100,00%	7	9	13	0,24%	57,20%
Trypsin precursor	TRYP_PIG	24.409,30	100,00%	7	8	35	0,65%	20,80%
Proline/betaine transporter (Proline por	sp|P0C0L7|PROP_ECOLI	54.848,50	100,00%	2	2	2	0,04%	5,60%
D-methionine-binding lipoprotein metQ pr	sp|P28635|METQ_ECOLI	29.431,90	100,00%	12	15	27	0,50%	53,50%
3-phenylpropionate	sp|P0ABR5|HCAE_ECOLI	51.109	97,80%	1	1	1	0,02%	2,21%

dioxygenase alpha sub		,70						
30S ribosomal protein S9.	sp|P0A7X3|RS9_ECOLI	14.725 ,40	100,00%	2	2	2	0,04%	15,50 %
Glycerol kinase (EC 2.7.1.30) (ATP:glyce	sp|P0A6F3|GLPK_ECOLI	56.100 ,10	100,00%	6	6	6	0,11%	14,40 %
Elongation factor Tu (EF-Tu) (P-43).	sp|P0A6N1|EFTU_ECOLI	43.182 ,60	100,00%	19	23	52	0,97%	56,20 %
Aerobic glycerol-3-phosphate dehydrogena	sp|P13035|GLPD_ECOLI	56.752 ,30	100,00%	7	8	10	0,19%	13,00 %
Bifunctional aspartokinase/homoserine de	sp|P00561|AK1H_ECOLI	89.121 ,90	99,80%	1	1	1	0,02%	1,59%
Keratin, type I cytoskeletal 10	K1C10_HUMAN	59.520 ,30	100,00%	3	3	3	0,06%	5,06%
Glycyl-tRNA synthetase alpha chain (EC 6	sp|P00960|SYGA_ECOLI	34.774 ,90	100,00%	4	4	4	0,07%	12,50 %
Phosphoglucosamine mutase (EC 5.4.2.10).	sp|P31120|GLMM_ECOLI	47.413 ,30	99,90%	2	2	2	0,04%	6,31%
3-oxoacyl-[acyl-carrier-protein] reducta	sp|P0AEK2|FABG_ECOLI	25.561 ,00	100,00%	10	22	39	0,73%	50,00 %
Delta-aminolevulinic acid dehydratase (E	sp|P0ACB2|HEM2_ECOLI	35.494 ,70	100,00%	2	2	2	0,04%	6,81%
Phosphoglycerate kinase (EC 2.7.2.3).	sp|P0A799|PGK_ECOLI	40.988 ,00	96,10%	1	1	1	0,02%	3,63%
Cell division protein zipA.	sp|P77173|ZIPA_ECOLI	36.474 ,50	99,90%	2	2	2	0,04%	9,76%
NADH-quinone oxidoreductase chain F (EC	sp|P31979|NUOF_ECOLI	49.294 ,50	96,10%	1	1	1	0,02%	2,92%
Putative HTH-type transcriptional regula	sp|P27434|YFGA_ECOLI	36.191 ,50	100,00%	2	2	2	0,04%	9,20%
Fructose-bisphosphate aldolase class II	sp|P0AB71|ALF_ECOLI	39.016 ,10	100,00%	5	5	7	0,13%	16,50 %
UTP--glucose-1-phosphate uridylyltransfe	sp|P0AAB6|GALF_ECOLI	32.830 ,10	96,10%	1	1	2	0,04%	4,04%
MltA-interacting protein precursor.	sp|P0A908|MIPA_ECOLI	27.831 ,90	100,00%	3	4	5	0,09%	16,50 %
Inner membrane lipoprotein yiaD precurso	sp|P37665|YIAD_ECOLI	22.197 ,60	100,00%	2	3	3	0,06%	16,40 %
Protein recA (Recombinase A).	sp|P0A7G6|RECA_ECOLI	37.843 ,50	100,00%	4	4	4	0,07%	14,80 %
ATP-dependent protease hslV (EC 3.4.25.-	sp|P0A7B8|HSLV_ECOLI	18.962 ,10	100,00%	5	5	8	0,15%	30,30 %
Long-chain fatty acid transport protein	sp|P10384|FADL_ECOLI	48.773 ,40	100,00%	3	3	3	0,06%	8,93%
ATP synthase B chain (EC 3.6.3.14).	sp|P0ABA0|ATPF_ECOLI	17.264 ,50	100,00%	12	15	19	0,36%	56,40 %
Hypothetical protein yraM.	sp|P45464|YRAM_ECOLI	72.825 ,80	100,00%	2	2	2	0,04%	3,54%
PTS system mannose-specific EIIAB compon	sp|P69797|PTNAB_ECOLI	34.916 ,20	100,00%	9	12	23	0,43%	26,10 %
Hypothetical UPF0070 protein yfgM.	sp|P76576|YFGM_ECOLI	22.176 ,40	100,00%	2	2	2	0,04%	21,40 %
Aldehyde-alcohol dehydrogenase [Includes	sp|P0A9Q7|ADHE_ECOLI	95.998 ,90	96,10%	1	1	1	0,02%	1,69%
50S ribosomal protein L1.	sp|P0A7L0|RL1_ECOLI	24.598 ,20	100,00%	5	6	6	0,11%	29,20 %
ATP-dependent protease La (EC 3.4.21.53)	sp|P0A9M0|LON_ECOLI	87.440 ,60	100,00%	9	9	11	0,21%	12,60 %
Small-conductance mechanosensitive chann	sp|P0C0S1|MSCS_ECOLI	30.897 ,00	100,00%	2	2	2	0,04%	8,04%

Protein rcsF.	sp|P69411|RCSF_ECOLI	14.163,50	96,10%	1	1	1	0,02%	10,40%
Hypothetical protein yggT.	sp|P64564|YGGT_ECOLI	21.168,60	99,90%	2	2	2	0,04%	11,20%
30S ribosomal protein S10.	sp|P0A7R5|RS10_ECOLI	11.735,80	100,00%	2	2	2	0,04%	23,30%
DNA gyrase subunit A (EC 5.99.1.3).	sp|P0AES4|GYRA_ECOLI	96.965,00	100,00%	16	16	18	0,34%	21,30%
Transport ATP-binding protein cydD.	sp|P29018|CYDD_ECOLI	65.058,60	100,00%	2	2	2	0,04%	4,25%
Transcription antitermination protein nu	sp|P0AFG0|NUSG_ECOLI	20.399,80	96,10%	1	1	1	0,02%	7,22%
Inner membrane transport protein ydhC.	sp|P37597|YDHC_ECOLI	43.368,60	96,10%	1	1	1	0,02%	3,47%
Alpha-ketoglutarate permease.	sp|P0AEX3|KGTP_ECOLI	47.054,00	96,10%	1	1	1	0,02%	2,55%
Acetyl-coenzyme A carboxylase carboxyl t	sp|P0A9Q5|ACCD_ECOLI	33.322,50	99,40%	1	1	1	0,02%	3,29%
50S ribosomal protein L9.	sp|P0A7R1|RL9_ECOLI	15.768,70	100,00%	2	2	2	0,04%	19,50%
PTS system trehalose-specific EIIBC comp	sp|P36672|PTTBC_ECOLI	51.082,60	100,00%	8	9	14	0,26%	14,60%
Antigen 43 precursor (AG43) (Fluffing pr	sp|P39180|AG43_ECOLI	106.823,60	100,00%	12	14	14	0,26%	16,90%
Tail-specific protease precursor (EC 3.4	sp|P23865|PRC_ECOLI	76.665,70	99,80%	1	1	1	0,02%	1,91%
Glucose-6-phosphate 1-dehydrogenase (EC	sp|P0AC53|G6PD_ECOLI	55.705,70	99,90%	1	1	1	0,02%	2,04%
Hypothetical protein yfdI.	sp|P76507|YFDI_ECOLI	51.487,90	100,00%	3	4	4	0,07%	7,45%
Aldehyde dehydrogenase A (EC 1.2.1.22) (	sp|P25553|ALDA_ECOLI	52.142,10	99,80%	2	2	2	0,04%	3,77%
Threonyl-tRNA synthetase (EC 6.1.1.3) (T	sp|P0A8M3|SYT_ECOLI	74.015,60	100,00%	9	9	9	0,17%	14,30%
30S ribosomal protein S7.	sp|P02359|RS7_ECOLI	19.888,10	99,20%	2	2	2	0,04%	14,60%
Glutamine transport system permease prot	sp|P0AEQ6|GLNP_ECOLI	24.365,90	99,80%	1	1	1	0,02%	5,02%
Multidrug resistance protein mdtB (Multi	sp|P76398|MDTB_ECOLI	112.081,10	98,00%	1	1	1	0,02%	0,96%
Prolyl-tRNA synthetase (EC 6.1.1.15) (Pr	sp|P16659|SYP_ECOLI	63.693,40	100,00%	3	3	3	0,06%	7,17%
30S ribosomal protein S13.	sp|P0A7S9|RS13_ECOLI	12.968,90	100,00%	5	5	5	0,09%	52,10%
Lysyl-tRNA synthetase (EC 6.1.1.6) (Lysi	sp|P0A8N3|SYK1_ECOLI	57.474,30	99,80%	1	1	1	0,02%	2,38%
Outer membrane protein X precursor.	sp|P0A917|OMPX_ECOLI	18.603,00	100,00%	5	9	12	0,22%	37,40%
Preprotein translocase secA subunit.	sp|P10408|SECA_ECOLI	102.025,30	100,00%	13	13	13	0,24%	16,60%
UDP-galactopyranose mutase (EC 5.4.99.9)	sp|P37747|GLF_ECOLI	42.967,60	99,90%	2	2	2	0,04%	5,72%
Bifunctional aspartokinase/homoserine de	sp|P00562|AK2H_ECOLI	88.757,50	99,70%	1	1	1	0,02%	0,99%
Enolase (EC 4.2.1.11) (2-phosphoglycerat	sp|P0A6P9|ENO_ECOLI	45.525,50	100,00%	8	8	9	0,17%	23,70%
Transcription termination factor rho (EC	sp|P0AG30|RHO_ECOLI	47.006,30	100,00%	2	2	2	0,04%	8,11%
3-octaprenyl-4-hydroxybenzoate carboxy-l	sp|P0AAB4|UBID_ECOLI	55.604,80	99,10%	1	1	1	0,02%	2,41%
Diaminopimelate decarboxylase (EC 4.1.1.	sp|P00861|DCDA_ECOLI	46.176,60	96,10%	1	1	1	0,02%	2,62%
Glycine dehydrogenase [decarboxylating]	sp|P33195|GCSP_ECOLI	104.246,10	100,00%	3	3	3	0,06%	3,77%

Hypothetical protein yibN.	sp|P0AG27|YIBN_ECOLI	15.596 ,20	100,00%	4	5	5	0,09%	32,90 %
Phosphoenolpyruvate-protein phosphotrans	sp|P08839|PT1_ECOLI	63.564 ,30	100,00%	4	4	4	0,07%	8,35%
50S ribosomal protein L24.	sp|P60624|RL24_ECOLI	11.185 ,10	96,10%	1	1	1	0,02%	9,71%
Low-affinity inorganic phosphate transpo	sp|P0AFJ7|PITA_ECOLI	53.390 ,90	99,10%	1	1	1	0,02%	2,00%
Gamma-glutamyl phosphate reductase (GPR)	sp|P07004|PROA_ECOLI	44.630 ,40	100,00%	7	7	9	0,17%	20,40 %
ATP-dependent Clp protease ATP-binding s	sp|P0A6H1|CLPX_ECOLI	46.226 ,10	100,00%	8	8	8	0,15%	25,10 %
Rod shape-determining protein mreB.	sp|P0A9X4|MREB_ECOLI	36.952 ,40	100,00%	6	6	6	0,11%	22,80 %
Cysteine synthase B (EC 2.5.1.47) (O-ace	sp|P16703|CYSM_ECOLI	32.664 ,60	95,80%	1	1	1	0,02%	4,29%
NADP-specific glutamate dehydrogenase (E	sp|P00370|DHE4_ECOLI	48.581 ,20	100,00%	7	7	7	0,13%	21,30 %
Small protein A precursor.	sp|P0A937|SMPA_ECOLI	12.302 ,00	100,00%	2	2	2	0,04%	26,50 %
Osmotically inducible lipoprotein E prec	sp|P0ADB1|OSME_ECOLI	12.020 ,50	100,00%	4	5	5	0,09%	29,50 %
50S ribosomal protein L7/L12 (L8).	sp|P0A7K2|RL7_ECOLI	12.163 ,80	100,00%	5	6	15	0,28%	36,70 %
Protein-export membrane protein secG (Pr	sp|P0AG99|SECG_ECOLI	11.365 ,80	100,00%	2	2	3	0,06%	27,30 %
Maltose transport system permease protei	sp|P02916|MALF_ECOLI	57.016 ,50	100,00%	2	2	2	0,04%	4,47%
Seryl-tRNA synthetase (EC 6.1.1.11) (Ser	sp|P0A8L1|SYS_ECOLI	48.414 ,80	100,00%	2	2	2	0,04%	4,88%
Polyribonucleotide nucleotidyltransferas	sp|P05055|PNP_ECOLI	77.101 ,00	100,00%	21	28	42	0,78%	38,50 %
Tyrosyl-tRNA synthetase (EC 6.1.1.1) (Ty	sp|P0AGJ9|SYY_ECOLI	47.397 ,00	100,00%	2	2	2	0,04%	6,15%
Chaperone surA precursor (Peptidyl-proly	sp|P0ABZ6|SURA_ECOLI	47.284 ,70	100,00%	5	5	6	0,11%	14,00 %
Protein ydgA precursor.	sp|P77804|YDGA_ECOLI	54.690 ,10	100,00%	7	7	7	0,13%	13,10 %
Putative frv operon regulatory protein [	sp|P32152|FRVR_ECOLI	66.002 ,40	97,90%	1	1	1	0,02%	1,20%
Periplasmic oligopeptide-binding protein	sp|P23843|OPPA_ECOLI	60.899 ,80	100,00%	8	10	11	0,21%	17,30 %
ATP synthase alpha chain (EC 3.6.3.14).	sp|P0ABB0|ATPA_ECOLI	55.223 ,20	100,00%	21	24	44	0,82%	48,90 %
Citrate synthase (EC 2.3.3.1).	sp|P0ABH7|CISY_ECOLI	48.016 ,80	100,00%	10	17	27	0,50%	28,80 %
3-hydroxydecanoyl-[acyl-carrier-protein]	sp|P0A6Q3|FABA_ECOLI	18.838 ,40	100,00%	5	5	8	0,15%	31,60 %
Cysteine desulfurase (EC 2.8.1.7) (Thil	sp|P0A6B7|ISCS_ECOLI	45.090 ,70	100,00%	2	2	2	0,04%	5,20%
Methionyl-tRNA synthetase (EC 6.1.1.10)	sp|P00959|SYM_ECOLI	76.125 ,50	100,00%	9	9	11	0,21%	15,20 %
Uridylate kinase (EC 2.7.4.-) (UK) (Urid	sp|P0A7E9|PYRH_ECOLI	25.839 ,70	96,10%	1	1	1	0,02%	4,58%
Chaperone clpB (Heat-shock protein F84.1	sp|P63284|CLPB_ECOLI	95.587 ,90	100,00%	22	28	35	0,65%	32,70 %
Outer membrane protein assembly factor y	sp|P0A940|YAET_ECOLI	90.554 ,40	100,00%	19	23	33	0,62%	30,70 %
Soluble pyridine nucleotide transhydroge	sp|P27306|STHA_ECOLI	51.429 ,80	96,80%	1	1	1	0,02%	1,51%
Translation initiation factor IF-2.	sp|P0A705|IF2_ECOLI	97.349 ,00	100,00%	21	23	26	0,49%	26,60 %

Pyruvate kinase I (EC 2.7.1.40) (PK-1).	sp|P0AD61|KPYK1_ECOLI	50.729,90	100,00%	12	14	18	0,34%	29,40%
Hypothetical ABC transporter ATP-binding	sp|P36879|YADG_ECOLI	34.648,40	100,00%	2	2	2	0,04%	6,49%
Nucleoside permease nupG (Nucleoside-tra	sp|P0AFF4|NUPG_ECOLI	46.391,50	99,90%	2	2	2	0,04%	4,55%
Multidrug resistance protein A.	sp|P27303|EMRA_ECOLI	42.736,10	99,90%	1	1	1	0,02%	3,85%
Major outer membrane lipoprotein precurs	sp|P69776|LPP_ECOLI	8.323,70	100,00%	6	13	67	1,25%	48,70%
Hypothetical protein yfgG.	sp|P64545|YFGG_ECOLI	7.461,70	96,10%	1	1	1	0,02%	23,80%
Bifunctional glmU protein [Includes: UDP	sp|P0ACC7|GLMU_ECOLI	49.190,50	100,00%	3	3	3	0,06%	7,24%
Hypothetical protein yoaF.	sp|P64493|YOAF_ECOLI	8.942,30	99,90%	1	1	1	0,02%	21,40%
50S ribosomal protein L28.	sp|P0A7M2|RL28_ECOLI	8.875,30	95,90%	1	1	1	0,02%	13,00%
Glucans biosynthesis protein D precursor	sp|P40120|OPGD_ECOLI	62.759,70	100,00%	2	2	2	0,04%	3,45%
Putative cytochrome d ubiquinol oxidase	sp|P0ADW3|YHCB_ECOLI	15.239,80	99,80%	1	1	2	0,04%	12,70%
Hypothetical protein yrbD precursor.	sp|P64604|YRBD_ECOLI	19.577,10	100,00%	3	3	3	0,06%	18,00%
Protein damX.	sp|P11557|DAMX_ECOLI	46.161,50	99,90%	3	3	3	0,06%	8,18%
Hypothetical lipoprotein yajG precursor.	sp|P0ADA5|YAJG_ECOLI	20.950,40	100,00%	4	5	11	0,21%	25,00%
Nucleoside-specific channel-forming prot	sp|P0A927|TSX_ECOLI	33.590,20	100,00%	2	2	6	0,11%	8,16%
Anaerobic C4-dicarboxylate transporter d	sp|P0ABN5|DCUA_ECOLI	45.752,20	100,00%	3	3	3	0,06%	6,93%
Protein tolB precursor.	sp|P0A855|TOLB_ECOLI	45.955,10	100,00%	7	8	9	0,17%	21,20%
Bifunctional putA protein [Includes: Pro	sp|P09546|PUTA_ECOLI	143.818,40	100,00%	7	7	7	0,13%	6,74%
Ribosome recycling factor (Ribosome-rele	sp|P0A805|RRF_ECOLI	20.639,30	100,00%	8	10	19	0,36%	44,30%
Protein yicC.	sp|P23839|YICC_ECOLI	33.175,20	100,00%	2	2	2	0,04%	8,71%
Ubiquinol oxidase polypeptide I (EC 1.10	sp|P0ABI8|CYOB_ECOLI	74.370,10	100,00%	5	7	12	0,22%	8,75%
Tyrosine-protein kinase wzc (EC 2.7.1.11	sp|P76387|WZC_ECOLI	79.345,10	100,00%	2	3	4	0,07%	3,61%
GTP-binding protein typA/BipA (Tyrosine	sp|P32132|TYPA_ECOLI	67.356,00	100,00%	5	6	6	0,11%	9,56%
ProP effector.	sp|P45577|PROQ_ECOLI	25.892,40	95,30%	1	1	1	0,02%	4,31%
L-fucose isomerase (EC 5.3.1.25) (6-deox	sp|P69922|FUCI_ECOLI	64.977,20	100,00%	4	5	5	0,09%	9,14%
UPF0092 protein yajC.	sp|P0ADZ7|YAJC_ECOLI	11.887,60	100,00%	4	6	12	0,22%	27,30%
Inner membrane protein yccF.	sp|P0AB12|YCCF_ECOLI	16.275,90	100,00%	2	2	2	0,04%	14,90%
Outer membrane protein A precursor (Oute	sp|P0A910|OMPA_ECOLI	37.201,00	100,00%	20	31	116	2,17%	46,50%
30S ribosomal protein S18.	sp|P0A7T7|RS18_ECOLI	8.855,80	100,00%	2	2	3	0,06%	29,70%
Probable formate transporter 1 (Formate	sp|P0AC23|FOCA_ECOLI	30.991,40	99,90%	1	1	1	0,02%	3,16%
Trigger factor (TF).	sp|P0A850|TIG_ECOLI	48.192,90	100,00%	6	7	9	0,17%	17,40%
Outer membrane protein F precursor (Pori	sp|P02931|OMPF_ECOLI	39.334,40	100,00%	2	2	2	0,04%	5,80%
Malate dehydrogenase (EC 1.1.1.37).	sp|P61889|MDH_ECOLI	32.337,30	100,00%	2	2	2	0,04%	6,09%

UPF0141 protein yijP.	sp|P32678|YIJP_ECOLI	66.612,50	99,90%	2	2	2	0,04%	5,72%
Hypothetical protein yehM.	sp|P33349|YEHM_ECOLI	83.392,30	95,90%	1	1	1	0,02%	1,05%
Transaldolase B (EC 2.2.1.2).	sp|P0A870|TALB_ECOLI	35.089,70	100,00%	5	5	5	0,09%	17,70%
Periplasmic beta-glucosidase precursor (	sp|P33363|BGLX_ECOLI	83.461,40	100,00%	6	6	6	0,11%	10,10%
Macrolide-specific ABC-type efflux carri	sp|P75831|MACB_ECOLI	70.702,90	99,60%	2	2	2	0,04%	4,78%
30S ribosomal protein S5.	sp|P0A7W1|RS5_ECOLI	17.471,80	100,00%	4	4	4	0,07%	38,00%
Ribonucleoside-diphosphate reductase 1 a	sp|P00452|RIR1_ECOLI	85.778,80	100,00%	4	4	4	0,07%	6,44%
Lipoprotein vacJ precursor.	sp|P76506|VACJ_ECOLI	28.042,30	100,00%	4	5	9	0,17%	15,50%
Outer membrane protein tolC precursor.	sp|P02930|TOLC_ECOLI	54.015,20	100,00%	9	12	12	0,22%	24,00%
Sec-independent protein translocase prot	sp|P69428|TATA_ECOLI	9.664,50	100,00%	2	2	2	0,04%	25,80%
ABC transporter periplasmic-binding prot	sp|P39325|YTFQ_ECOLI	34.345,60	100,00%	2	2	3	0,06%	8,18%
Putative sensor-like histidine kinase yf	sp|P52101|YFHK_ECOLI	55.700,20	95,90%	1	1	1	0,02%	2,42%
Vitamin B12 transporter btuB precursor (	sp|P06129|BTUB_ECOLI	68.408,20	100,00%	7	7	10	0,19%	15,10%
Cytochrome d ubiquinol oxidase subunit I	sp|P0ABK2|CYDB_ECOLI	42.454,10	100,00%	3	7	10	0,19%	7,65%
Septum site-determining protein minD (Ce	sp|P0AEZ3|MIND_ECOLI	29.484,10	96,10%	1	1	1	0,02%	5,20%
Magnesium and cobalt transport protein c	sp|P0ABI4|CORA_ECOLI	36.591,40	100,00%	5	6	8	0,15%	18,70%
PTS system glucose-specific EIICB compon	sp|P69786|PTGCB_ECOLI	50.678,40	100,00%	13	16	39	0,73%	29,80%
NADH-quinone oxidoreductase chain C/D (E	sp|P33599|NUOCD_ECOLI	68.696,40	100,00%	5	5	8	0,15%	8,17%
60 kDa chaperonin (Protein Cpn60) (groEL	sp|P0A6F5|CH60_ECOLI	57.197,70	100,00%	25	34	78	1,46%	56,70%
Protein hflC (EC 3.4.-.-).	sp|P0ABC3|HFLC_ECOLI	37.650,90	100,00%	5	6	7	0,13%	18,60%
Cytochrome d ubiquinol oxidase subunit I	sp|P0ABJ9|CYDA_ECOLI	58.207,40	100,00%	16	20	34	0,64%	27,40%
Inner membrane protein yjgP.	sp|P0AF98|YJGP_ECOLI	40.358,90	100,00%	3	3	3	0,06%	8,47%

86

87

Table S2. Proteins identified by Mass spectroscopy in the Native gel fragment of interest excized from stationary phase lane (control)

Protein name	Protein accession numbers	Protein molecular weight (Da)	Protein identification probability	Exclusive unique peptide count	Exclusive unique spectrum count	Total spectrum count	Percentage of total spectra	Percentage sequence coverage
Maltose/maltodextrin import ATP-binding	sp|P68187|MALK_ECOLI	40.989,90	100,00%	7	9	16	0,29%	22,10%
Hydroxylamine reductase (EC 1.7.-.-) (Hy	sp|P75825|HCP_ECOLI	60.065,50	99,70%	1	1	1	0,02%	1,64%
Phosphate-binding periplasmic protein pr	sp|P0AG82|PSTS_ECOLI	37.024,70	100,00%	10	10	12	0,22%	39,30%
Outer-membrane lipoprotein lolB precurso	sp|P61320|LOLB_ECOLI	23.551,60	96,30%	1	1	1	0,02%	5,31%
Chaperone protein dnaK (Heat shock prote	sp|P0A6Y8|DNAK_ECOLI	68.984,90	100,00%	19	22	26	0,48%	37,20%
Mannose permease IID component (PTS syst	sp|P69805|PTND_ECOLI	31.304,00	100,00%	8	10	16	0,29%	32,50%
Hypothetical protein yqjD.	sp|P64581|YQJD_ECOLI	11.051,70	100,00%	2	3	5	0,09%	25,70%
Aminoacyl-histidine dipeptidase (EC 3.4.	sp|P15288|PEPD_ECOLI	52.785,30	100,00%	7	8	10	0,18%	17,10%
D-ribose-binding periplasmic protein pre	sp|P02925|RBSB_ECOLI	30.950,50	100,00%	13	31	86	1,58%	53,40%
Rod shape-determining protein mreC.	sp|P16926|MREC_ECOLI	39.530,60	96,30%	1	1	1	0,02%	3,54%
Preprotein translocase secY subunit.	sp|P0AGA2|SECY_ECOLI	48.514,00	100,00%	2	3	3	0,06%	5,87%
PTS system maltose- and glucose-specific	sp|P19642|PTOCB_ECOLI	56.628,40	96,70%	1	1	1	0,02%	2,26%
Fumarate hydratase class I, aerobic (EC	sp|P0AC33|FUMA_ECOLI	60.169,10	100,00%	4	4	4	0,07%	10,60%
Low-specificity L-threonine aldolase (EC	sp|P75823|LTAE_ECOLI	36.495,40	100,00%	2	2	2	0,04%	7,51%
Uronate isomerase (EC 5.3.1.12) (Glucuro	sp|P0A8G3|UXAC_ECOLI	53.989,00	99,70%	1	1	1	0,02%	2,13%
Hypothetical protein yjeI precursor.	sp|P0AF70|YJEI_ECOLI	11.958,30	99,90%	1	1	2	0,04%	16,20%
NADH-quinone oxidoreductase chain G (EC	sp|P33602|NUOG_ECOLI	100.168,30	100,00%	8	9	10	0,18%	9,70%
Aconitate hydratase 2 (EC 4.2.1.3) (Citr	sp|P36683|ACON2_ECOLI	93.501,00	100,00%	13	13	18	0,33%	18,50%
Protein ycaC.	sp|P21367|YCAC_ECOLI	23.100,90	100,00%	4	6	9	0,17%	22,60%
Trypsin precursor	TRYP_PIG	24.409,30	100,00%	6	7	30	0,55%	16,50%
50S ribosomal protein L7/L12 (L8).	sp|P0A7K2|RL7_ECOLI	12.163,80	100,00%	5	6	11	0,20%	36,70%
Hypothetical UPF0135 protein ybgI.	sp|P0AFP6|YBGI_ECOLI	26.892,70	100,00%	2	4	4	0,07%	14,60%
Hypothetical protein yefG (GalF transfer	sp|P37749|YEFG_ECOLI	37.759,10	100,00%	1	1	1	0,02%	3,33%
Succinyl-CoA synthetase beta chain (EC 6	sp|P0A836|SUCC_ECOLI	41.392,70	100,00%	17	24	34	0,62%	49,50%
Hypothetical protein yiaF.	sp|P0ADK0|YIAF_ECOLI	30.158,60	100,00%	5	6	6	0,11%	24,60%
Protein ygaU.	sp|P0ADE6|YGAU_ECOLI	15.932,20	100,00%	3	3	3	0,06%	23,00%
50S ribosomal protein L22.	sp|P61175|RL22_ECOLI	12.226,60	100,00%	2	2	2	0,04%	20,90%
Fumarate hydratase class II (EC 4.2.1.2)	sp|P05042|FUMC_ECOLI	50.489,50	95,20%	1	1	1	0,02%	3,85%
Serine hydroxymethyltransferase (EC 2.1.	sp|P0A825|GLYA_ECOLI	45.316,70	100,00%	4	5	6	0,11%	11,00%

6-phosphogluconate dehydrogenase, decarb	sp|P00350|6PGD_ECOLI	51.483,20	100,00%	9	9	9	0,17%	28,20%
Pyrroline-5-carboxylate reductase (EC 1.	sp|P0A9L8|PROC_ECOLI	28.145,00	100,00%	5	8	12	0,22%	24,50%
Protein yhdH.	sp|P26646|YHDH_ECOLI	34.723,50	100,00%	2	2	2	0,04%	6,48%
Cation/acetate symporter actP (Acetate t	sp|P32705|ACTP_ECOLI	59.200,00	99,90%	2	2	2	0,04%	3,83%
Tripeptide permease tppB.	sp|P77304|TPPB_ECOLI	53.993,30	96,30%	1	1	1	0,02%	2,60%
Serine transporter.	sp|P0AAD6|SDAC_ECOLI	46.909,00	100,00%	4	5	6	0,11%	9,56%
Succinate dehydrogenase flavoprotein sub	sp|P0AC41|DHSA_ECOLI	64.421,90	100,00%	3	3	3	0,06%	5,95%
Pyruvate kinase I (EC 2.7.1.40) (PK-1).	sp|P0AD61|KPYK1_ECOLI	50.729,90	100,00%	16	18	29	0,53%	37,40%
Adenylate kinase (EC 2.7.4.3) (ATP-AMP t	sp|P69441|KAD_ECOLI	23.586,40	100,00%	4	4	6	0,11%	22,00%
Peroxiredoxin osmC (EC 1.11.1.15) (Osmot	sp|P0C0L2|OSMC_ECOLI	14.957,30	99,80%	1	1	1	0,02%	13,40%
Maltose-binding periplasmic protein prec	sp|P0AEX9|MALE_ECOLI	43.389,90	100,00%	13	15	22	0,40%	41,90%
Valyl-tRNA synthetase (EC 6.1.1.9) (Vali	sp|P07118|SYV_ECOLI	108.195,30	100,00%	5	5	5	0,09%	7,26%
Succinate-semialdehyde dehydrogenase [NA	sp|P25526|GABD_ECOLI	51.721,60	100,00%	4	4	4	0,07%	10,60%
Periplasmic dipeptide transport protein	sp|P23847|DPPA_ECOLI	60.294,60	100,00%	6	7	9	0,17%	13,60%
GMP synthase [glutamine-hydrolyzing] (EC	sp|P04079|GUAA_ECOLI	58.679,20	100,00%	1	1	1	0,02%	2,67%
Lipoprotein 34 precursor.	sp|P0A903|NLPB_ECOLI	36.842,30	96,30%	1	1	1	0,02%	5,52%
Protease VII precursor (EC 3.4.23.-) (Om	sp|P09169|OMPT_ECOLI	35.563,90	100,00%	6	6	8	0,15%	22,40%
Cytochrome o ubiquinol oxidase subunit I	sp|P0ABJ3|CYOC_ECOLI	22.623,20	96,30%	1	2	3	0,06%	5,39%
ATP synthase C chain (EC 3.6.3.14) (Lipi	sp|P68699|ATPL_ECOLI	8.256,70	99,80%	2	2	4	0,07%	20,30%
2,3,4,5-tetrahydropyridine-2,6-dicarboxy	sp|P0A9D8|DAPD_ECOLI	29.891,60	100,00%	5	5	7	0,13%	18,60%
Adenylosuccinate synthetase (EC 6.3.4.4)	sp|P0A7D4|PURA_ECOLI	47.214,50	100,00%	9	9	10	0,18%	22,30%
GTP pyrophosphokinase (EC 2.7.6.5) (ATP:	sp|P0AG20|RELA_ECOLI	83.876,30	99,70%	1	1	1	0,02%	1,88%
2-dehydro-3-deoxyphosphooctonate aldolas	sp|P0A715|KDSA_ECOLI	30.832,90	98,30%	2	2	2	0,04%	5,99%
Alpha-galactosidase (EC 3.2.1.22) (Melib	sp|P06720|AGAL_ECOLI	50.657,80	99,90%	2	3	3	0,06%	4,21%
Cell division protein ftsZ.	sp|P0A9A6|FTSZ_ECOLI	40.324,00	100,00%	12	13	14	0,26%	38,90%
Glucosamine--fructose-6-phosphate aminot	sp|P17169|GLMS_ECOLI	66.764,50	100,00%	5	5	7	0,13%	8,88%
KHG/KDPG aldolase [Includes: 4-hydroxy-2	sp|P0A955|ALKH_ECOLI	22.284,30	100,00%	2	2	2	0,04%	15,00%
Tagatose-1,6-bisphosphate aldolase gatY	sp|P37192|GATY_ECOLI	30.811,50	100,00%	6	7	16	0,29%	22,20%
Fructose-bisphosphate aldolase class I (	sp|P0A991|ALF1_ECOLI	37.978,90	96,30%	1	1	1	0,02%	6,02%
Probable ABC transporter ATP-binding pro	sp|P0A9V1|YHBG_ECOLI	26.670,30	100,00%	3	3	4	0,07%	16,20%
Purine nucleoside phosphorylase deoD-typ	sp|P0ABP8|DEOD_ECOLI	25.819,30	100,00%	4	4	7	0,13%	18,50%
2-oxoglutarate dehydrogenase E1 componen	sp|P0AFG3|ODO1_ECOLI	105.061,90	100,00%	3	3	3	0,06%	3,32%
Galactoside transport system permease pr	sp|P23200|MGLC_ECOLI	35.551,90	96,30%	1	1	2	0,04%	3,87%
Putative xanthine dehydrogenase yagR, mo	sp|P77489|YAGR_ECOLI	78.089,00	95,30%	1	1	2	0,04%	1,23%
Cystine-binding periplasmic	sp|P0AEM9|FLIY_ECOLI	29.039,9	100,00%	3	3	3	0,06%	10,20%

protein prec		0						
Phosphoenolpyruvate carboxylase (EC 4.1.	sp|P00864|CAPP_ECOLI	99.066,00	100,00%	12	12	14	0,26%	13,70%
Acetate kinase (EC 2.7.2.1) (Acetokinase	sp|P0A6A3|ACKA_ECOLI	43.291,50	96,30%	1	1	1	0,02%	3,75%
Protein csiD.	sp|P76621|CSID_ECOLI	37.360,80	100,00%	4	4	5	0,09%	16,00%
Hypothetical protein yicH.	sp|P31433|YICH_ECOLI	62.274,00	96,30%	1	1	1	0,02%	1,76%
Protein ygfZ.	sp|P0ADE8|YGFZ_ECOLI	35.963,60	96,10%	1	1	1	0,02%	3,38%
Biotin carboxylase (EC 6.3.4.14) (A subu	sp|P24182|ACCC_ECOLI	49.321,90	100,00%	6	6	10	0,18%	14,90%
10 kDa chaperonin (Protein Cpn10) (groES	sp|P0A6F9|CH10_ECOLI	10.387,00	100,00%	2	2	2	0,04%	24,70%
Hypothetical adenine-specific methylase	sp|P39199|YFCB_ECOLI	35.002,40	100,00%	2	2	2	0,04%	6,13%
Phosphoheptose isomerase (EC 5.3.1.-) (S	sp|P63224|GMHA_ECOLI	20.815,40	100,00%	2	2	2	0,04%	10,40%
2,3-bisphosphoglycerate-independent phos	sp|P37689|GPMI_ECOLI	56.194,50	96,30%	1	1	1	0,02%	2,72%
Probable glutamate/gamma-aminobutyrate a	sp|P63235|GADC_ECOLI	55.078,90	100,00%	3	3	4	0,07%	7,05%
Tryptophanase (EC 4.1.99.1) (L-tryptopha	sp|P0A853|TNAA_ECOLI	52.774,80	100,00%	26	45	141	2,59%	56,50%
Aspartyl-tRNA synthetase (EC 6.1.1.12) (	sp|P21889|SYD_ECOLI	65.915,50	100,00%	8	8	10	0,18%	15,10%
Formate acetyltransferase 1 (EC 2.3.1.54	sp|P09373|PFLB_ECOLI	85.229,10	100,00%	18	24	28	0,51%	32,10%
Hypothetical lipoprotein yedD precursor.	sp|P31063|YEDD_ECOLI	14.983,50	100,00%	3	4	5	0,09%	31,40%
CTP synthase (EC 6.3.4.2) (UTP--ammonia	sp|P0A7E5|PYRG_ECOLI	60.243,40	100,00%	2	2	2	0,04%	4,41%
6-phosphofructokinase isozyme I (EC 2.7.	sp|P0A796|K6PF1_ECOLI	34.843,00	100,00%	2	3	3	0,06%	8,44%
50S ribosomal protein L5.	sp|P62399|RL5_ECOLI	20.171,50	100,00%	4	5	5	0,09%	25,30%
50S ribosomal protein L6.	sp|P0AG55|RL6_ECOLI	18.772,30	95,30%	1	1	1	0,02%	6,82%
Glucosamine-6-phosphate deaminase (EC 3.	sp|P0A759|NAGB_ECOLI	29.774,40	100,00%	2	2	2	0,04%	8,65%
Phenylalanyl-tRNA synthetase alpha chain	sp|P08312|SYFA_ECOLI	36.832,50	99,30%	1	1	1	0,02%	3,67%
Protein hflC (EC 3.4.-.-).	sp|P0ABC3|HFLC_ECOLI	37.650,90	94,90%	1	1	1	0,02%	5,09%
Putative metalloprotease yggG (EC 3.4.24	sp|P25894|YGGG_ECOLI	26.842,40	100,00%	2	2	2	0,04%	10,70%
Glyceraldehyde-3-phosphate dehydrogenase	sp|P0A9B2|G3P1_ECOLI	35.401,40	100,00%	17	23	38	0,70%	53,60%
Biopolymer transport exbB protein.	sp|P0ABU7|EXBB_ECOLI	26.287,80	99,80%	1	1	1	0,02%	6,15%
Protein-export membrane protein secF.	sp|P0AG93|SECF_ECOLI	35.383,50	96,30%	1	1	1	0,02%	4,02%
UDP-glucose 4-epimerase (EC 5.1.3.2) (Ga	sp|P09147|GALE_ECOLI	37.265,10	96,30%	1	1	1	0,02%	5,62%
Cysteine synthase B (EC 2.5.1.47) (O-ace	sp|P16703|CYSM_ECOLI	32.664,60	99,60%	1	1	1	0,02%	4,29%
Enoyl-[acyl-carrier-protein] reductase [	sp|P0AEK4|FABI_ECOLI	27.733,40	100,00%	2	2	2	0,04%	8,81%
Acyl-CoA thioester hydrolase ybgC precur	sp|P46130|YBHC_ECOLI	46.081,90	100,00%	6	6	8	0,15%	18,00%
Chaperone protein htpG (Heat shock prote	sp|P0A6Z3|HTPG_ECOLI	71.425,00	100,00%	3	3	3	0,06%	6,25%
Hypothetical protein yeaG.	sp|P0ACY3|YEAG_ECOLI	74.483,00	99,90%	1	1	1	0,02%	2,02%
Glycogen phosphorylase (EC 2.4.1.1).	sp|P0AC86|PHSG_ECOLI	93.175,30	100,00%	4	4	4	0,07%	6,01%
Alkyl hydroperoxide reductase subunit C	sp|P0AE08|AHPC_ECOLI	20.630,70	100,00%	7	11	16	0,29%	43,00%

Protease degQ precursor (EC 3.4.21.-).	sp|P39099|DEGQ_ECOLI	47.207,10	95,30%	1	1	1	0,02%	2,42%
Aminopeptidase N (EC 3.4.11.2) (Alpha-am	sp|P04825|AMPN_ECOLI	98.790,60	100,00%	4	4	5	0,09%	5,18%
Colicin I receptor precursor.	sp|P17315|CIRA_ECOLI	73.896,60	96,30%	1	1	1	0,02%	1,81%
Alkyl hydroperoxide reductase subunit F	sp|P35340|AHPF_ECOLI	56.178,20	100,00%	2	2	3	0,06%	4,41%
Glycerol-3-phosphate transporter (G-3-P	sp|P08194|GLPT_ECOLI	50.312,50	100,00%	2	2	2	0,04%	5,09%
Chain length determinant protein (Polysa	sp|P76372|WZZB_ECOLI	36.455,60	100,00%	4	4	4	0,07%	15,30%
Isocitrate dehydrogenase [NADP] (EC 1.1.	sp|P08200|IDH_ECOLI	45.758,00	100,00%	4	4	6	0,11%	13,50%
Probable protease htpX (EC 3.4.24.-) (He	sp|P23894|HTPX_ECOLI	31.924,10	96,30%	1	1	1	0,02%	3,75%
50S ribosomal protein L18.	sp|P0C018|RL18_ECOLI	12.769,70	95,50%	1	1	1	0,02%	7,69%
Transketolase 1 (EC 2.2.1.1) (TK 1).	sp|P27302|TKT1_ECOLI	72.213,00	100,00%	10	11	14	0,26%	17,60%
Outer membrane lipoprotein slyB precurso	sp|P0A905|SLYB_ECOLI	15.600,60	100,00%	4	6	9	0,17%	38,70%
50S ribosomal protein L17.	sp|P0AG44|RL17_ECOLI	14.365,20	100,00%	3	3	4	0,07%	26,00%
Xaa-Pro dipeptidase (EC 3.4.13.9) (X-Pro	sp|P21165|PEPQ_ECOLI	50.177,30	99,00%	1	1	1	0,02%	2,26%
Pyruvate dehydrogenase E1 component (EC	sp|P0AFG8|ODP1_ECOLI	99.540,00	100,00%	6	6	6	0,11%	7,56%
Phosphoenolpyruvate-protein phosphotrans	sp|P37177|PT1P_ECOLI	83.718,50	100,00%	3	3	3	0,06%	5,21%
Ribose transport ATP-binding protein rbs	sp|P04983|RBSA_ECOLI	55.043,30	99,90%	1	1	1	0,02%	2,59%
Rare lipoprotein A precursor.	sp|P10100|RLPA_ECOLI	37.527,60	100,00%	2	2	2	0,04%	6,91%
Magnesium transporting ATPase, P-type 1	sp|P0ABB8|ATMA_ECOLI	99.468,80	100,00%	3	3	3	0,06%	5,23%
Hypothetical protein yibN.	sp|P0AG27|YIBN_ECOLI	15.596,20	100,00%	2	2	2	0,04%	17,50%
Transcription elongation protein nusA (N	sp|P0AFF6|NUSA_ECOLI	54.871,40	100,00%	2	2	2	0,04%	5,05%
2-amino-3-ketobutyrate coenzyme A ligase	sp|P0AB77|KBL_ECOLI	43.117,70	100,00%	7	7	7	0,13%	20,40%
NADH-quinone oxidoreductase chain B (EC	sp|P0AFC7|NUOB_ECOLI	25.055,60	100,00%	4	4	6	0,11%	16,80%
Aldehyde dehydrogenase B (EC 1.2.1.-).	sp|P37685|ALDB_ECOLI	56.307,40	100,00%	7	9	10	0,18%	18,90%
Isoleucyl-tRNA synthetase (EC 6.1.1.5) (	sp|P00956|SYI_ECOLI	104.167,10	100,00%	7	7	8	0,15%	7,36%
Lipoprotein yfgL precursor.	sp|P77774|YFGL_ECOLI	41.886,90	100,00%	4	4	4	0,07%	13,00%
ADP-L-glycero-D-manno-heptose-6-epimeras	sp|P67910|HLDD_ECOLI	34.894,30	100,00%	8	8	9	0,17%	29,00%
Transketolase 2 (EC 2.2.1.1) (TK 2).	sp|P33570|TKT2_ECOLI	73.044,10	99,70%	2	2	2	0,04%	2,70%
Inner membrane protein yjgQ.	sp|P0ADC6|YJGQ_ECOLI	39.620,10	100,00%	2	2	2	0,04%	6,39%
Inner membrane protein oxaA.	sp|P25714|OXAA_ECOLI	61.528,00	100,00%	2	2	2	0,04%	4,56%
EIImtl	6H-C384S-W42,6H-W42,EII-F97W,sp|P00550|PTM3C_ECOLI	67.857,70	100,00%	8	8	9	0,17%	15,00%
30S ribosomal protein S1.	sp|P0AG67|RS1_ECOLI	61.157,40	100,00%	16	23	30	0,55%	31,40%
Na(+)/H(+) antiporter 2 (Sodium/proton a	sp|P0AFA7|NHAB_ECOLI	56.730,50	96,30%	1	1	1	0,02%	2,14%
Elongation factor G (EF-G).	sp|P0A6M8|EFG_ECOLI	77.450,80	100,00%	22	30	47	0,86%	43,50%
Mannose permease IIC component (PTS syst	sp|P69801|PTNC_ECOLI	27.636,40	100,00%	2	3	6	0,11%	11,70%

Hypothetical zinc-type alcohol dehydroge	sp|P27250|YJGB_ECOLI	36.501,80	100,00%	2	2	2	0,04%	7,37%
30S ribosomal protein S16.	sp|P0A7T3|RS16_ECOLI	9.190,60	99,70%	1	1	1	0,02%	13,40%
Protein-export membrane protein secD.	sp|P0AG90|SECD_ECOLI	66.634,50	100,00%	14	14	19	0,35%	28,00%
Spermidine/putrescine import ATP-binding	sp|P69874|POTA_ECOLI	43.028,40	100,00%	3	3	3	0,06%	9,52%
Protein ydgH precursor.	sp|P76177|YDGH_ECOLI	33.903,30	100,00%	2	2	2	0,04%	8,60%
GTP cyclohydrolase I (EC 3.5.4.16) (GTP-	sp|P0A6T5|GCH1_ECOLI	24.699,80	100,00%	3	3	3	0,06%	15,80%
Oligopeptidase A (EC 3.4.24.70).	sp|P27298|OPDA_ECOLI	77.169,50	99,50%	2	2	2	0,04%	3,53%
Organic solvent tolerance protein precur	sp|P31554|OSTA_ECOLI	89.672,80	100,00%	12	13	16	0,29%	17,10%
Aspartate aminotransferase (EC 2.6.1.1)	sp|P00509|AAT_ECOLI	43.574,10	100,00%	10	11	13	0,24%	28,00%
Superoxide dismutase [Mn] (EC 1.15.1.1)	sp|P00448|SODM_ECOLI	22.966,60	100,00%	4	4	6	0,11%	20,00%
Transport ATP-binding protein cydC.	sp|P23886|CYDC_ECOLI	62.922,80	99,90%	1	1	1	0,02%	2,62%
Cell division protein ftsY.	sp|P10121|FTSY_ECOLI	54.512,50	100,00%	2	2	2	0,04%	4,23%
Alanyl-tRNA synthetase (EC 6.1.1.7) (Ala	sp|P00957|SYA_ECOLI	96.033,60	100,00%	31	39	55	1,01%	43,40%
Succinyl-CoA synthetase alpha chain (EC	sp|P0AGE9|SUCD_ECOLI	29.646,60	100,00%	11	14	21	0,39%	46,90%
Hypothetical protein ycfM.	sp|P0AB38|YCFM_ECOLI	22.515,20	100,00%	4	4	5	0,09%	29,60%
Adenylosuccinate lyase (EC 4.3.2.2) (Ade	sp|P0AB89|PUR8_ECOLI	51.544,10	100,00%	8	8	10	0,18%	19,30%
Hypothetical protein ydbA.	sp|P33666|YDBA_ECOLI	205.947,30	98,00%	1	1	1	0,02%	0,70%
Ribokinase (EC 2.7.1.15).	sp|P0A9J6|RBSK_ECOLI	32.290,60	100,00%	3	3	3	0,06%	12,00%
Xaa-Pro aminopeptidase (EC 3.4.11.9) (X-	sp|P15034|AMPP_ECOLI	49.684,60	100,00%	6	6	6	0,11%	14,10%
Glucose-6-phosphate isomerase (EC 5.3.1.	sp|P0A6T1|G6PI_ECOLI	61.531,40	100,00%	9	9	11	0,20%	20,20%
Rare lipoprotein B precursor.	sp|P0ADC1|RLPB_ECOLI	21.357,30	100,00%	5	6	9	0,17%	31,60%
L-threonine 3-dehydrogenase (EC 1.1.1.10	sp|P07913|TDH_ECOLI	37.239,60	100,00%	3	3	3	0,06%	12,30%
Protein-export protein secB.	sp|P0AG86|SECB_ECOLI	17.277,20	99,80%	1	1	1	0,02%	9,68%
Hypothetical lipoprotein ydcL precursor.	sp|P64451|YDCL_ECOLI	24.427,30	100,00%	9	11	16	0,29%	54,50%
Fumarate reductase flavoprotein subunit	sp|P00363|FRDA_ECOLI	65.840,40	100,00%	5	5	5	0,09%	8,82%
Outer membrane protein C precursor (Pori	sp|P06996|OMPC_ECOLI	40.369,00	100,00%	4	4	4	0,07%	13,40%
Elongation factor Ts (EF-Ts).	sp|P0A6P1|EFTS_ECOLI	30.291,60	100,00%	11	12	14	0,26%	36,90%
NAD(P) transhydrogenase subunit beta (EC	sp|P0AB67|PNTB_ECOLI	48.724,00	100,00%	8	13	16	0,29%	22,90%
Superoxide dismutase [Fe] (EC 1.15.1.1).	sp|P0AGD3|SODF_ECOLI	21.135,00	100,00%	4	4	7	0,13%	25,50%
Stringent starvation protein A.	sp|P0ACA3|SSPA_ECOLI	24.174,60	100,00%	2	2	2	0,04%	11,80%
Spermidine/putrescine-binding periplasmi	sp|P0AFK9|POTD_ECOLI	38.868,90	100,00%	2	2	2	0,04%	7,47%
Leucyl-tRNA synthetase (EC 6.1.1.4) (Leu	sp|P07813|SYL_ECOLI	97.234,30	100,00%	2	2	2	0,04%	2,79%
2,3-bisphosphoglycerate-dependent phosph	sp|P62707|GPMA_ECOLI	28.426,10	100,00%	4	5	7	0,13%	18,10%
Putative HTH-type transcriptional regula	sp|P77634|YBCM_ECOLI	30.842,00	99,10%	1	1	1	0,02%	4,15%
50S ribosomal protein L10 (50S ribosomal	sp|P0A7J3|RL10_ECOLI	17.580,60	100,00%	8	9	12	0,22%	53,70%

30S ribosomal protein S4.	sp|P0A7V8|RS4_ECOLI	23.338,50	100,00%	3	3	4	0,07%	20,00%
Peroxidase/catalase HPI (EC 1.11.1.6) (C	sp|P13029|CATA_ECOLI	80.025,80	100,00%	22	29	45	0,83%	32,90%
Ribose transport system permease protein	sp|P0AGI1|RBSC_ECOLI	33.452,90	100,00%	2	2	3	0,06%	6,54%
Asparaginyl-tRNA synthetase (EC 6.1.1.22	sp|P0A8M0|SYN_ECOLI	52.439,10	100,00%	8	8	8	0,15%	20,90%
Glucokinase (EC 2.7.1.2) (Glucose kinase	sp|P0A6V8|GLK_ECOLI	34.723,90	100,00%	3	3	3	0,06%	10,90%
Ubiquinol oxidase polypeptide II precurs	sp|P0ABJ1|CYOA_ECOLI	34.912,30	100,00%	6	9	21	0,39%	26,30%
Cold shock-like protein cspC (CSP-C).	sp|P0A9Y6|CSPC_ECOLI	7.271,10	100,00%	2	2	3	0,06%	42,60%
S-adenosylmethionine synthetase (EC 2.5.	sp|P0A817|METK_ECOLI	41.820,90	100,00%	2	2	2	0,04%	7,83%
Maltoporin precursor (Maltose-inducible	sp|P02943|LAMB_ECOLI	49.912,80	100,00%	5	7	10	0,18%	14,80%
3-oxoacyl-[acyl-carrier-protein] synthas	sp|P0A953|FABB_ECOLI	42.613,40	100,00%	4	5	6	0,11%	10,30%
Peptidyl-prolyl cis-trans isomerase D (E	sp|P0ADY1|PPID_ECOLI	68.151,40	100,00%	13	13	15	0,28%	27,60%
NADH dehydrogenase (EC 1.6.99.3).	sp|P00393|DHNA_ECOLI	47.228,80	99,30%	1	1	1	0,02%	2,54%
Hypothetical protein yneA precursor.	sp|P76142|YNEA_ECOLI	36.685,00	100,00%	3	3	3	0,06%	9,71%
30S ribosomal protein S2.	sp|P0A7V0|RS2_ECOLI	26.613,10	100,00%	5	5	5	0,09%	24,20%
Hypothetical lipoprotein yifL precursor.	sp|P0ADN6|YIFL_ECOLI	7.177,40	96,30%	1	1	2	0,04%	34,30%
Phospho-N-acetylmuramoyl-pentapeptide-tr	sp|P0A6W3|MRAY_ECOLI	39.875,60	99,70%	1	1	1	0,02%	2,22%
UPF0304 protein yfbU.	sp|P0A8W8|YFBU_ECOLI	19.536,50	100,00%	2	2	2	0,04%	17,10%
UPF0169 lipoprotein yfiO precursor.	sp|P0AC02|YFIO_ECOLI	27.830,30	100,00%	4	4	4	0,07%	20,40%
Phosphocarrier protein HPr (Histidine-co	sp|P0AA04|PTHP_ECOLI	9.119,20	99,90%	1	1	1	0,02%	15,30%
Phenylalanyl-tRNA synthetase beta chain	sp|P07395|SYFB_ECOLI	87.378,60	100,00%	3	3	3	0,06%	4,91%
Anthranilate synthase component I (EC 4.	sp|P00895|TRPE_ECOLI	57.495,80	100,00%	3	3	3	0,06%	6,54%
Pyruvate dehydrogenase [cytochrome] (EC	sp|P07003|POXB_ECOLI	62.012,00	100,00%	3	3	3	0,06%	6,64%
Low affinity tryptophan permease.	sp|P23173|TNAB_ECOLI	45.213,70	100,00%	2	2	3	0,06%	5,54%
D-3-phosphoglycerate dehydrogenase (EC 1	sp|P0A9T0|SERA_ECOLI	44.045,90	100,00%	2	2	2	0,04%	6,36%
Succinate dehydrogenase iron-sulfur prot	sp|P07014|DHSB_ECOLI	26.771,00	100,00%	2	2	2	0,04%	10,10%
Sec-independent protein translocase prot	sp|P69428|TATA_ECOLI	9.664,50	99,90%	1	1	1	0,02%	14,60%
Entericidin B precursor.	sp|P0ADB7|ECNB_ECOLI	4.809,50	96,30%	1	2	4	0,07%	39,60%
Uridine phosphorylase (EC 2.4.2.3) (UrdP	sp|P12758|UDP_ECOLI	27.027,50	100,00%	6	8	10	0,18%	27,80%
NAD(P) transhydrogenase subunit alpha (E	sp|P07001|PNTA_ECOLI	54.623,70	100,00%	9	14	22	0,40%	23,70%
Probable formate transporter 1 (Formate	sp|P0AC23|FOCA_ECOLI	30.991,40	100,00%	3	4	4	0,07%	9,12%
Nucleoside permease nupC (Nucleoside-tra	sp|P0AFF2|NUPC_ECOLI	43.477,70	100,00%	2	2	3	0,06%	5,00%
Peptidoglycan-associated lipoprotein pre	sp|P0A912|PAL_ECOLI	18.824,50	100,00%	4	4	7	0,13%	29,50%
Putative betaine aldehyde dehydrogenase	sp|P77674|YDCW_ECOLI	50.829,80	100,00%	7	9	11	0,20%	21,30%
Protein dcrB precursor.	sp|P0AEE1|DCRB_ECOLI	19.787,70	100,00%	2	2	2	0,04%	16,20%
Lysyl-tRNA synthetase, heat inducible (E	sp|P0A8N5|SYK2_ECOLI	57.697,30	100,00%	2	2	2	0,04%	9,13%

D-methionine-binding lipoprotein metQ pr	sp|P28635|METQ_ECOLI	29.431,90	100,00%	6	6	6	0,11%	30,60%
Transaldolase A (EC 2.2.1.2).	sp|P0A867|TALA_ECOLI	35.659,50	99,50%	1	1	1	0,02%	5,06%
Glutathione reductase (EC 1.8.1.7) (GR)	sp|P06715|GSHR_ECOLI	48.772,60	100,00%	2	2	2	0,04%	4,89%
Putative transport protein yidE.	sp|P60872|YIDE_ECOLI	58.941,10	99,90%	1	1	1	0,02%	1,99%
Glycerol kinase (EC 2.7.1.30) (ATP:glyce	sp|P0A6F3|GLPK_ECOLI	56.100,10	100,00%	5	5	6	0,11%	13,00%
Elongation factor Tu (EF-Tu) (P-43).	sp|P0A6N1|EFTU_ECOLI	43.182,60	100,00%	21	29	56	1,03%	61,10%
Aspartate ammonia-lyase (EC 4.3.1.1) (As	sp|P0AC38|ASPA_ECOLI	52.356,30	100,00%	16	20	31	0,57%	39,30%
Hypothetical UPF0070 protein yfgM.	sp|P76576|YFGM_ECOLI	22.176,40	100,00%	2	2	2	0,04%	21,40%
Inner membrane protein yccF.	sp|P0AB12|YCCF_ECOLI	16.275,90	100,00%	2	2	2	0,04%	14,90%
High affinity ribose transport protein r	sp|P04982|RBSD_ECOLI	15.292,20	100,00%	3	3	4	0,07%	24,50%
Glycyl-tRNA synthetase alpha chain (EC 6	sp|P00960|SYGA_ECOLI	34.774,90	100,00%	4	5	7	0,13%	21,50%
3-oxoacyl-[acyl-carrier-protein] reducta	sp|P0AEK2|FABG_ECOLI	25.561,00	100,00%	7	12	17	0,31%	43,00%
Delta-aminolevulinic acid dehydratase (E	sp|P0ACB2|HEM2_ECOLI	35.494,70	100,00%	6	6	8	0,15%	21,70%
Phosphoglycerate kinase (EC 2.7.2.3).	sp|P0A799|PGK_ECOLI	40.988,00	100,00%	8	9	11	0,20%	23,30%
NADH-quinone oxidoreductase chain F (EC	sp|P31979|NUOF_ECOLI	49.294,50	100,00%	7	7	7	0,13%	20,90%
Fructose-bisphosphate aldolase class II	sp|P0AB71|ALF_ECOLI	39.016,10	100,00%	8	11	15	0,28%	29,90%
UTP--glucose-1-phosphate uridylyltransfe	sp|P0AAB6|GALF_ECOLI	32.830,10	96,30%	1	1	2	0,04%	4,04%
MltA-interacting protein precursor.	sp|P0A908|MIPA_ECOLI	27.831,90	96,30%	1	1	2	0,04%	5,24%
Aconitate hydratase 1 (EC 4.2.1.3) (Citr	sp|P25516|ACON1_ECOLI	97.517,60	100,00%	3	3	3	0,06%	3,03%
Protein recA (Recombinase A).	sp|P0A7G6|RECA_ECOLI	37.843,50	100,00%	5	5	5	0,09%	19,60%
ATP-dependent protease hslV (EC 3.4.25.-	sp|P0A7B8|HSLV_ECOLI	18.962,10	100,00%	2	2	2	0,04%	10,30%
Hypothetical UPF0325 protein yaeH.	sp|P62768|YAEH_ECOLI	15.097,00	99,90%	2	2	2	0,04%	18,80%
Long-chain fatty acid transport protein	sp|P10384|FADL_ECOLI	48.773,40	96,30%	1	1	1	0,02%	2,68%
ATP synthase B chain (EC 3.6.3.14).	sp|P0ABA0|ATPF_ECOLI	17.264,50	100,00%	9	11	19	0,35%	46,80%
Outer membrane protein X precursor.	sp|P0A917|OMPX_ECOLI	18.603,00	98,80%	2	2	2	0,04%	13,50%
ATP-dependent Clp protease proteolytic s	sp|P0A6G7|CLPP_ECOLI	23.187,30	100,00%	2	3	4	0,07%	14,50%
Hypothetical protein yraM.	sp|P45464|YRAM_ECOLI	72.825,80	100,00%	5	5	5	0,09%	7,67%
PTS system mannose-specific EIIAB compon	sp|P69797|PTNAB_ECOLI	34.916,20	100,00%	14	23	38	0,70%	42,50%
ATP-dependent hsl protease ATP-binding s	sp|P0A6H5|HSLU_ECOLI	49.595,20	99,60%	2	2	2	0,04%	5,42%
Cysteine synthase A (EC 2.5.1.47) (O-ace	sp|P0ABK5|CYSK_ECOLI	34.359,70	100,00%	10	11	16	0,29%	38,80%
Catalase HPII (EC 1.11.1.6) (Hydroxypero	sp|P21179|CATE_ECOLI	84.165,10	100,00%	22	27	38	0,70%	30,40%
50S ribosomal protein L1.	sp|P0A7L0|RL1_ECOLI	24.598,20	100,00%	6	7	9	0,17%	33,90%
Thiosulfate-binding protein precursor.	sp|P16700|CYSP_ECOLI	37.615,40	100,00%	3	3	3	0,06%	9,47%
Protein rcsF.	sp|P69411|RCSF_ECOLI	14.163,50	95,90%	1	1	1	0,02%	10,40%
30S ribosomal protein S10.	sp|P0A7R5|RS10_ECOLI	11.735,8	100,00%	2	2	2	0,04%	23,30%

		0						
Triosephosphate isomerase (EC 5.3.1.1) (	sp|P0A858|TPIS_ECOLI	26.971,8 0	100,00%	4	4	4	0,07%	18,00%
ATP synthase beta chain (EC 3.6.3.14).	sp|P0ABB4|ATPB_ECOLI	50.194,3 0	100,00%	5	5	5	0,09%	16,30%
DNA gyrase subunit A (EC 5.99.1.3).	sp|P0AES4|GYRA_ECOLI	96.965,0 0	100,00%	13	13	14	0,26%	18,70%
Phosphoserine aminotransferase (EC 2.6.1	sp|P23721|SERC_ECOLI	39.652,9 0	100,00%	2	2	2	0,04%	7,76%
Alpha-ketoglutarate permease.	sp|P0AEX3|KGTP_ECOLI	47.054,0 0	100,00%	2	2	2	0,04%	5,09%
ATP synthase alpha chain (EC 3.6.3.14).	sp|P0ABB0|ATPA_ECOLI	55.223,2 0	100,00%	10	11	11	0,20%	22,40%
50S ribosomal protein L9.	sp|P0A7R1|RL9_ECOLI	15.768,7 0	100,00%	3	3	3	0,06%	23,50%
PTS system trehalose-specific EIIBC comp	sp|P36672|PTTBC_ECOLI	51.082,6 0	100,00%	5	6	9	0,17%	10,40%
Antigen 43 precursor (AG43) (Fluffing pr	sp|P39180|AG43_ECOLI	106.823, 60	100,00%	25	39	64	1,17%	34,40%
Tail-specific protease precursor (EC 3.4	sp|P23865|PRC_ECOLI	76.665,7 0	100,00%	9	10	12	0,22%	13,60%
ABC transporter ATP-binding protein yjjK	sp|P0A9W3|YJJK_ECOLI	62.314,2 0	100,00%	2	2	2	0,04%	3,97%
Branched-chain-amino-acid aminotransfera	sp|P0AB80|ILVE_ECOLI	33.962,6 0	96,30%	1	1	1	0,02%	5,19%
Glucose-6-phosphate 1-dehydrogenase (EC	sp|P0AC53|G6PD_ECOLI	55.705,7 0	99,70%	2	2	2	0,04%	4,28%
Hypothetical protein yfdI.	sp|P76507|YFDI_ECOLI	51.487,9 0	100,00%	1	2	3	0,06%	2,71%
Aldehyde dehydrogenase A (EC 1.2.1.22) (	sp|P25553|ALDA_ECOLI	52.142,1 0	100,00%	15	20	39	0,72%	37,00%
Lysine-arginine-ornithine-binding peripl	sp|P09551|ARGT_ECOLI	27.993,1 0	95,60%	1	1	1	0,02%	2,69%
Glutamine transport system permease prot	sp|P0AEQ6|GLNP_ECOLI	24.365,9 0	95,00%	1	1	1	0,02%	4,11%
Prolyl-tRNA synthetase (EC 6.1.1.15) (Pr	sp|P16659|SYP_ECOLI	63.693,4 0	100,00%	10	11	14	0,26%	20,80%
30S ribosomal protein S13.	sp|P0A7S9|RS13_ECOLI	12.968,9 0	100,00%	2	2	2	0,04%	26,50%
Lysyl-tRNA synthetase (EC 6.1.1.6) (Lysi	sp|P0A8N3|SYK1_ECOLI	57.474,3 0	100,00%	5	5	5	0,09%	10,90%
Lipid A export ATP-binding/permease prot	sp|P60752|MSBA_ECOLI	64.462,7 0	100,00%	10	10	12	0,22%	20,30%
Inner membrane protein yqjE.	sp|P64585|YQJE_ECOLI	15.147,6 0	100,00%	2	2	2	0,04%	13,40%
Maltose transport system permease protei	sp|P02916|MALF_ECOLI	57.016,5 0	100,00%	6	6	8	0,15%	16,90%
Glucose-1-phosphate adenylyltransferase	sp|P0A6V1|GLGC_ECOLI	48.566,9 0	96,30%	1	1	1	0,02%	2,33%
D-lactate dehydrogenase (EC 1.1.1.28) (R	sp|P06149|DLD_ECOLI	64.482,9 0	100,00%	5	5	5	0,09%	8,95%
Tryptophan synthase beta chain (EC 4.2.1	sp|P0A879|TRPB_ECOLI	42.852,8 0	100,00%	2	2	2	0,04%	9,60%
Enolase (EC 4.2.1.11) (2-phosphoglycerat	sp|P0A6P9|ENO_ECOLI	45.525,5 0	100,00%	11	14	25	0,46%	34,60%
Malate synthase G (EC 2.3.3.9) (MSG).	sp|P37330|MASZ_ECOLI	80.358,9 0	100,00%	11	11	13	0,24%	19,30%
Glutamate decarboxylase beta (EC 4.1.1.1	sp|P69910|DCEB_ECOLI	52.669,6 0	100,00%	18	26	51	0,94%	44,00%
Phosphoenolpyruvate carboxykinase [ATP]	sp|P22259|PPCK_ECOLI	59.645,3 0	100,00%	5	5	7	0,13%	13,00%
UPF0076 protein yjgF.	sp|P0AF93|YJGF_ECOLI	13.480,2 0	99,90%	2	2	2	0,04%	17,30%
Diaminopimelate decarboxylase (EC 4.1.1.	sp|P00861|DCDA_ECOLI	46.176,6 0	100,00%	7	9	11	0,20%	27,90%
2-hydroxy-3-oxopropionate reductase (EC	sp|P0ABQ2|GARR_ECOLI	30.428,3 0	100,00%	3	3	4	0,07%	15,30%
Single-strand binding protein (SSB) (Hel	sp|P0AGE0|SSB_ECOLI	18.843,2 0	96,30%	1	1	1	0,02%	7,91%

Glycine dehydrogenase [decarboxylating]	sp|P33195|GCSP_ECOLI	104.246,10	100,00%	10	10	12	0,22%	13,20%
Outer membrane protein slp precursor.	sp|P37194|SLP_ECOLI	20.964,10	100,00%	3	3	3	0,06%	20,20%
Phosphoenolpyruvate-protein phosphotrans	sp|P08839|PT1_ECOLI	63.564,30	100,00%	7	8	9	0,17%	13,90%
Glutamate decarboxylase alpha (EC 4.1.1.	sp|P69908|DCEA_ECOLI	52.686,80	96,30%	1	1	1	0,02%	44,00%
ATP-dependent Clp protease ATP-binding s	sp|P0A6H1|CLPX_ECOLI	46.226,10	100,00%	2	2	2	0,04%	6,38%
Rod shape-determining protein mreB.	sp|P0A9X4|MREB_ECOLI	36.952,40	99,80%	1	1	1	0,02%	4,03%
Hypothetical GST-like protein yghU.	sp|Q46845|YGHU_ECOLI	32.392,20	100,00%	2	2	2	0,04%	8,68%
NADP-specific glutamate dehydrogenase (E	sp|P00370|DHE4_ECOLI	48.581,20	100,00%	12	15	18	0,33%	36,00%
Small protein A precursor.	sp|P0A937|SMPA_ECOLI	12.302,00	100,00%	2	2	2	0,04%	32,70%
Protein grpE (HSP-70 cofactor) (Heat sho	sp|P09372|GRPE_ECOLI	21.797,80	96,30%	1	1	1	0,02%	7,11%
Osmotically inducible lipoprotein E prec	sp|P0ADB1|OSME_ECOLI	12.020,50	100,00%	5	9	12	0,22%	37,50%
Protein-export membrane protein secG (Pr	sp|P0AG99|SECG_ECOLI	11.365,80	100,00%	2	2	3	0,06%	27,30%
Seryl-tRNA synthetase (EC 6.1.1.11) (Ser	sp|P0A8L1|SYS_ECOLI	48.414,80	100,00%	6	6	8	0,15%	16,50%
Glucose-1-phosphatase precursor (EC 3.1.	sp|P19926|AGP_ECOLI	45.682,90	100,00%	6	6	7	0,13%	17,40%
Glutamate-1-semialdehyde 2,1-aminomutase	sp|P23893|GSA_ECOLI	45.366,30	100,00%	3	3	3	0,06%	9,86%
30S ribosomal protein S6 [Contains: 30S	sp|P02358|RS6_ECOLI	15.703,30	96,30%	1	1	1	0,02%	8,15%
Bifunctional protein hldE [Includes: D-b	sp|P76658|HLDE_ECOLI	51.050,60	99,50%	1	1	1	0,02%	2,52%
Polyribonucleotide nucleotidyltransferas	sp|P05055|PNP_ECOLI	77.101,00	100,00%	20	26	34	0,62%	36,40%
Tyrosyl-tRNA synthetase (EC 6.1.1.1) (Ty	sp|P0AGJ9|SYY_ECOLI	47.397,00	100,00%	2	2	2	0,04%	6,15%
D-galactose-binding periplasmic protein	sp|P0AEE5|DGAL_ECOLI	35.713,30	100,00%	9	11	16	0,29%	34,90%
Isocitrate lyase (EC 4.1.3.1) (Isocitras	sp|P0A9G6|ACEA_ECOLI	47.522,00	100,00%	13	16	19	0,35%	47,70%
Chaperone surA precursor (Peptidyl-proly	sp|P0ABZ6|SURA_ECOLI	47.284,70	100,00%	4	4	5	0,09%	12,10%
Protein ydgA precursor.	sp|P77804|YDGA_ECOLI	54.690,10	100,00%	7	7	7	0,13%	18,30%
Hypothetical lipoprotein ygdI precursor.	sp|P65292|YGDI_ECOLI	8.174,30	96,30%	1	2	2	0,04%	26,70%
Periplasmic oligopeptide-binding protein	sp|P23843|OPPA_ECOLI	60.899,80	100,00%	23	34	61	1,12%	47,10%
Citrate synthase (EC 2.3.3.1).	sp|P0ABH7|CISY_ECOLI	48.016,80	100,00%	24	33	74	1,36%	61,80%
3-hydroxydecanoyl-[acyl-carrier-protein]	sp|P0A6Q3|FABA_ECOLI	18.838,40	99,70%	2	2	3	0,06%	11,70%
4-aminobutyrate aminotransferase (EC 2.6	sp|P22256|GABT_ECOLI	45.775,60	100,00%	5	5	6	0,11%	15,50%
Methionyl-tRNA synthetase (EC 6.1.1.10)	sp|P00959|SYM_ECOLI	76.125,50	100,00%	4	4	4	0,07%	7,84%
Outer membrane protein assembly factor y	sp|P0A940|YAET_ECOLI	90.554,40	100,00%	18	21	25	0,46%	31,50%
Translation initiation factor IF-2.	sp|P0A705|IF2_ECOLI	97.349,00	100,00%	3	3	3	0,06%	3,26%
Putative Na(+)/H(+) exchanger yjcE.	sp|P32703|YJCE_ECOLI	60.525,40	97,90%	1	1	1	0,02%	2,55%
NAD-dependent malic enzyme (EC 1.1.1.38)	sp|P26616|MAO1_ECOLI	63.198,80	100,00%	10	10	10	0,18%	19,50%
Hypothetical ABC transporter ATP-binding	sp|P36879|YADG_ECOLI	34.648,40	100,00%	2	2	2	0,04%	7,47%
D-amino acid dehydrogenase	sp|P0A6J5|DADA_ECOLI	47.607,6	100,00%	4	4	4	0,07%	10,40%

small subunit		0						
Transaldolase B (EC 2.2.1.2).	sp|P0A870|TALB_ECOLI	35.089,7 0	100,00%	14	15	20	0,37%	58,90%
Biosynthetic arginine decarboxylase (EC	sp|P21170|SPEA_ECOLI	73.899,0 0	100,00%	9	9	10	0,18%	16,40%
Thiol peroxidase (EC 1.11.1.-) (Scavenga	sp|P0A862|TPX_ECOLI	17.704,0 0	96,30%	1	1	2	0,04%	7,78%
Major outer membrane lipoprotein precurs	sp|P69776|LPP_ECOLI	8.323,70	100,00%	5	12	49	0,90%	48,70%
Phosphopentomutase (EC 5.4.2.7) (Phospho	sp|P0A6K6|DEOB_ECOLI	44.371,1 0	96,30%	1	1	1	0,02%	2,95%
Trigger factor (TF).	sp|P0A850|TIG_ECOLI	48.192,9 0	100,00%	10	12	15	0,28%	26,60%
50S ribosomal protein L28.	sp|P0A7M2|RL28_ECOLI	8.875,30	99,90%	2	2	3	0,06%	13,00%
Putative cytochrome d ubiquinol oxidase	sp|P0ADW3|YHCB_ECOLI	15.239,8 0	100,00%	6	11	16	0,29%	63,40%
Hypothetical lipoprotein yajG precursor.	sp|P0ADA5|YAJG_ECOLI	20.950,4 0	100,00%	4	4	9	0,17%	25,00%
Putative aldolase yneB (EC 4.2.1.-).	sp|P76143|YNEB_ECOLI	31.893,0 0	100,00%	16	23	42	0,77%	59,10%
Ribulose-phosphate 3-epimerase (EC 5.1.3	sp|P0AG07|RPE_ECOLI	24.554,9 0	96,30%	1	1	1	0,02%	6,67%
Nucleoside-specific channel-forming prot	sp|P0A927|TSX_ECOLI	33.590,2 0	100,00%	2	2	6	0,11%	8,16%
Anaerobic C4-dicarboxylate transporter d	sp|P0ABN5|DCUA_ECOLI	45.752,2 0	100,00%	3	3	4	0,07%	7,16%
Phosphoglucomutase (EC 5.4.2.2) (Glucose	sp|P36938|PGM_ECOLI	58.361,6 0	100,00%	3	3	3	0,06%	6,59%
Protein tolB precursor.	sp|P0A855|TOLB_ECOLI	45.955,1 0	100,00%	4	4	4	0,07%	11,40%
Bifunctional putA protein [Includes: Pro	sp|P09546|PUTA_ECOLI	143.818, 40	100,00%	21	23	29	0,53%	19,50%
Ribosome recycling factor (Ribosome-rele	sp|P0A805|RRF_ECOLI	20.639,3 0	100,00%	7	8	11	0,20%	40,00%
UTP--glucose-1-phosphate uridylyltransfe	sp|P0AEP3|GALU_ECOLI	32.810,8 0	96,30%	1	1	1	0,02%	4,32%
Protein yicC.	sp|P23839|YICC_ECOLI	33.175,2 0	100,00%	3	4	4	0,07%	13,60%
Ubiquinol oxidase polypeptide I (EC 1.10	sp|P0ABI8|CYOB_ECOLI	74.370,1 0	100,00%	7	11	16	0,29%	11,50%
NADP-dependent L-serine/L-allo-threonine	sp|P39831|YDFG_ECOLI	27.248,7 0	96,30%	1	1	1	0,02%	7,26%
D-serine/D-alanine/glycine transporter.	sp|P0AAE0|CYCA_ECOLI	51.660,8 0	96,30%	1	1	1	0,02%	2,98%
Tyrosine-protein kinase wzc (EC 2.7.1.11	sp|P76387|WZC_ECOLI	79.345,1 0	100,00%	2	3	5	0,09%	3,61%
GTP-binding protein typA/BipA (Tyrosine	sp|P32132|TYPA_ECOLI	67.356,0 0	100,00%	2	2	2	0,04%	3,46%
ProP effector.	sp|P45577|PROQ_ECOLI	25.892,4 0	96,30%	1	1	1	0,02%	4,31%
Galactoside transport ATP-binding protei	sp|P0AAG8|MGLA_ECOLI	56.417,7 0	96,30%	1	1	1	0,02%	2,57%
L-fucose isomerase (EC 5.3.1.25) (6-deox	sp|P69922|FUCI_ECOLI	64.977,2 0	100,00%	2	2	2	0,04%	3,21%
UPF0092 protein yajC.	sp|P0ADZ7|YAJC_ECOLI	11.887,6 0	100,00%	4	4	10	0,18%	27,30%
Flavoprotein wrbA (Trp repressor binding	sp|P0A8G6|WRBA_ECOLI	20.714,2 0	98,00%	1	1	1	0,02%	8,63%
Outer membrane protein A precursor (Oute	sp|P0A910|OMPA_ECOLI	37.201,0 0	100,00%	15	20	42	0,77%	42,80%
Hypothetical lipoprotein yddW precursor.	sp|P64426|YDDW_ECOLI	49.575,1 0	99,80%	1	1	2	0,04%	2,51%
30S ribosomal protein S18.	sp|P0A7T7|RS18_ECOLI	8.855,80	100,00%	2	2	3	0,06%	24,30%
Inner membrane protein ybaL.	sp|P39830|YBAL_ECOLI	59.425,9 0	96,30%	1	1	1	0,02%	3,05%
Outer membrane protein F precursor (Pori	sp|P02931|OMPF_ECOLI	39.334,4 0	100,00%	7	8	11	0,20%	20,70%

Malate dehydrogenase (EC 1.1.1.37).	sp|P61889|MDH_ECOLI	32.337,30	100,00%	11	15	28	0,51%	38,10%
Trehalose-6-phosphate hydrolase (EC 3.2.	sp|P28904|TREC_ECOLI	63.838,50	100,00%	2	2	2	0,04%	3,81%
50S ribosomal protein L3.	sp|P60438|RL3_ECOLI	22.242,80	95,60%	1	1	1	0,02%	4,78%
Aspartate-semialdehyde dehydrogenase (EC	sp|P0A9Q9|DHAS_ECOLI	40.018,60	100,00%	2	2	3	0,06%	8,99%
Periplasmic beta-glucosidase precursor (	sp|P33363|BGLX_ECOLI	83.461,40	100,00%	3	3	3	0,06%	4,71%
Inorganic pyrophosphatase (EC 3.6.1.1) (	sp|P0A7A9|IPYR_ECOLI	19.572,80	100,00%	3	3	3	0,06%	16,00%
Glycyl-tRNA synthetase beta chain (EC 6.	sp|P00961|SYGB_ECOLI	76.684,10	100,00%	23	30	44	0,81%	39,70%
Starvation-sensing protein rspA.	sp|P38104|RSPA_ECOLI	45.969,40	99,00%	2	2	2	0,04%	9,16%
30S ribosomal protein S5.	sp|P0A7W1|RS5_ECOLI	17.471,80	100,00%	5	6	6	0,11%	48,20%
Hypothetical protein yhiI precursor.	sp|P37626|YHII_ECOLI	38.828,50	100,00%	2	2	2	0,04%	7,89%
Sensor protein kdpD (EC 2.7.3.-).	sp|P21865|KDPD_ECOLI	98.719,90	98,60%	2	2	2	0,04%	3,24%
Ribonucleoside-diphosphate reductase 1 a	sp|P00452|RIR1_ECOLI	85.778,80	100,00%	3	3	3	0,06%	5,65%
Lysophospholipase L2 (EC 3.1.1.5) (Lecit	sp|P07000|PLDB_ECOLI	38.978,50	95,00%	1	1	1	0,02%	5,59%
Hypothetical UPF0142 protein ycaO.	sp|P75838|YCAO_ECOLI	65.653,60	100,00%	2	2	2	0,04%	3,92%
Phosphoenolpyruvate synthase (EC 2.7.9.2	sp|P23538|PPSA_ECOLI	87.304,40	99,80%	2	2	2	0,04%	3,41%
Outer membrane protein tolC precursor.	sp|P02930|TOLC_ECOLI	54.015,20	96,30%	1	1	1	0,02%	3,23%
HTH-type transcriptional regulator malT	sp|P06993|MALT_ECOLI	103.121,50	100,00%	3	3	3	0,06%	3,88%
ABC transporter periplasmic-binding prot	sp|P39325|YTFQ_ECOLI	34.345,60	100,00%	7	10	19	0,35%	29,20%
Vitamin B12 transporter btuB precursor (	sp|P06129|BTUB_ECOLI	68.408,20	100,00%	7	7	7	0,13%	14,80%
Cytochrome d ubiquinol oxidase subunit I	sp|P0ABK2|CYDB_ECOLI	42.454,10	100,00%	3	7	11	0,20%	7,65%
Septum site-determining protein minD (Ce	sp|P0AEZ3|MIND_ECOLI	29.484,10	100,00%	3	3	3	0,06%	14,90%
PTS system glucose-specific EIICB compon	sp|P69786|PTGCB_ECOLI	50.678,40	100,00%	8	11	20	0,37%	20,80%
D-lactate dehydrogenase (EC 1.1.1.28) (D	sp|P52643|LDHD_ECOLI	36.535,70	100,00%	2	2	2	0,04%	7,60%
NADH-quinone oxidoreductase chain C/D (E	sp|P33599|NUOCD_ECOLI	68.696,40	100,00%	14	16	20	0,37%	26,80%
Cytochrome d ubiquinol oxidase subunit I	sp|P0ABJ9|CYDA_ECOLI	58.207,40	100,00%	19	26	57	1,05%	30,10%
Inner membrane protein yjgP.	sp|P0AF98|YJGP_ECOLI	40.358,90	100,00%	3	3	3	0,06%	10,40%
Carbon starvation protein A.	sp|P15078|CSTA_ECOLI	75.107,10	100,00%	6	8	11	0,20%	8,27%
Pyruvate kinase II (EC 2.7.1.40) (PK-2).	sp|P21599|KPYK2_ECOLI	51.226,60	100,00%	13	15	22	0,40%	30,90%
Peptidase B (EC 3.4.11.23) (Aminopeptida	sp|P37095|PEPB_ECOLI	46.181,80	100,00%	8	9	11	0,20%	23,20%

92

93

Supplemental text.

To further investigate the role of tags fused to cell division proteins we compared the effects of mild overexpression of GFP-fusions to FtsQ and FtsQ D237N to that of untagged FtsQ and FtsQ D237N. FtsQ, GFP-FtsQ, FtsQ D237N and GFP-FtsQ D237N were expressed at endogenous levels in *E. coli* MC4100 in addition to the chromosomal *ftsQ* and subsequently dilutions of these cultures were spotted on a plate and incubated to form colonies. GFP-FtsQ and GFP-FtsQ D237N are both expressed at endogenous levels from a leaky $Trc99_{down}$ promotor, FtsQ and FtsQ D237N are both expressed at endogenous levels from a pBAD promotor after induction with 0.2% arabinose (Fig. S5B). A clear difference was observed between strains expressing FtsQ D237N and GFP-FtsQ D237N. As previously shown, mild overexpression of FtsQ D237N disrupts the 1 MDa cell division complex. Here, mild expression of FtsQ D237N and of GFP-FtsQ D237N results in less growth on the plate as compared to empty vector. GFP-FtsQ D237N is always expressed and thus this strain is inhibited in growth by one order of magnitude as compared to empty vector. FtsQ-D237N is expressed after induction with 0.2% arabinose. When not induced, this strain grows similar to empty vector; when FtsQ D237N is induced, growth is inhibited by two orders of magnitude as compared to empty vector and by one order of magnitude as compared to GFP-FtsQ D237N (Fig. S5A). Thus, addition of a GFP-tag to the mutant FtsQ D237N makes it less inhibitory on growth. Also, a difference in growth can be observed between strains expressing additional FtsQ and GFP-FtsQ. Here, mild expression of GFP-FtsQ results in less growth. GFP-FtsQ is always expressed and thus this strain is inhibited in growth by one order of magnitude as compared to empty vector. FtsQ is expressed after induction with 0.2% arabinose. Whether induced or not, this strain grows similar as compared to empty vector (Fig. 5A). Thus, addition of a GFP-tag to the mutant FtsQ D237N makes the mutant less growth-inhibiting, whereas addition of a GFP-tag to wild type FtsQ makes it more growth-

inhibiting. This observation is in agreement with the finding that GFP-FtsQ protein complexes at a different size than 1 MDa (Fig. 5).

Supplementary reference

1. Wagner, S. *et al*. Consequences of membrane protein overexpression in *Escherichia coli*. *Mol. Cell. Proteomics* **6**, 1527-1550 (2007).

Supplementary file S1 Raw data of the Mass Spectrometry experiment. The data can be viewed with the freely available demo version of the Scaffold proteome software package (http://www.proteomesoftware.com/products/scaffold/)
